# Nuclear actin polymerization rapidly mediates replication fork remodeling upon stress by limiting PrimPol activity

**DOI:** 10.1038/s41467-023-43183-5

**Published:** 2023-11-28

**Authors:** Maria Dilia Palumbieri, Chiara Merigliano, Daniel González-Acosta, Danina Kuster, Jana Krietsch, Henriette Stoy, Thomas von Känel, Svenja Ulferts, Bettina Welter, Joël Frey, Cyril Doerdelmann, Andrea Sanchi, Robert Grosse, Irene Chiolo, Massimo Lopes

**Affiliations:** 1 Institute of Molecular Cancer Research, University of Zurich, Zurich, Switzerland; 2 Molecular and Computational Biology Department, University of Southern California, Los Angeles, CA USA; 3 Institute of Experimental and Clinical Pharmacology and Toxicology, Medical Faculty, University of Freiburg, Freiburg im Breisgau, Germany; 4 CIBSS - Centre for Integrative Biological Signaling Studies, University of Freiburg, Freiburg im Breisgau, Germany; 5 Present Address: Department of Cellular and Molecular Medicine, Copenhagen University, Copenhagen, Denmark

**Keywords:** Stalled forks, DNA damage response

## Abstract

Cells rapidly respond to replication stress actively slowing fork progression and inducing fork reversal. How replication fork plasticity is achieved in the context of nuclear organization is currently unknown. Using nuclear actin probes in living and fixed cells, we visualized nuclear actin filaments in unperturbed S phase and observed their rapid extension in number and length upon genotoxic treatments, frequently taking contact with replication factories. Chemically or genetically impairing nuclear actin polymerization shortly before these treatments prevents active fork slowing and abolishes fork reversal. Defective fork remodeling is linked to deregulated chromatin loading of PrimPol, which promotes unrestrained and discontinuous DNA synthesis and limits the recruitment of RAD51 and SMARCAL1 to nascent DNA. Moreover, defective nuclear actin polymerization upon mild replication interference induces chromosomal instability in a PRIMPOL-dependent manner. Hence, by limiting PrimPol activity, nuclear F-actin orchestrates replication fork plasticity and is a key molecular determinant in the rapid cellular response to genotoxic treatments.

## Abstract (web summary)

How nuclear architecture assists the replication stress response is still largely unknown. Here the authors show that nuclear actin polymerization rapidly extends upon mild DNA damage. By limiting Primpol activity, this response mediates fork slowing and reversal, protecting chromosome stability.

## Introduction

Interference with the DNA replication process (i.e. replication stress, RS) can be induced by numerous endogenous and exogenous sources^1^ and has recently emerged as a key molecular determinant of genomic instability in early tumorigenesis^2^. Moreover, as tumor cells experience high endogenous levels of RS, additional replication interference by genotoxic treatments or inactivation of key players of the RS response represent promising strategies for cancer chemotherapy^3,4^. Although the RS response is frequently studied upon conditions of severe fork stalling – e.g. by nucleotide depletion or extensive DNA damage – it is crucial to unravel the specific responses to mild replication interference to investigate tumorigenesis and improve therapeutic perspectives, as these conditions are more likely to reflect clinically relevant RS levels.

A key emerging aspect of the RS response in human cells is the plasticity of replication fork architecture^5^. This entails complex unwinding and annealing reactions of DNA strands at replication forks challenged by DNA lesions or other RS sources, and frequently leads to their conversion into 4-way junctions, so-called reversed forks^6^. This transaction promotes an active slowdown of replication fork progression and allows for different DNA damage tolerance mechanisms, which overall stabilize stalled forks and promote cellular resistance to genotoxic treatments^5–7^. Several specialized factors mediate this transaction, including the DNA translocases SMARCAL1, ZRANB3 and HLTF^8–10^, and the central recombinase RAD51^11,12^. However, reversed forks are also intrinsically unstable intermediates and in certain genetic backgrounds may trigger unscheduled nucleolytic degradation of nascent DNA, contributing to chemosensitivity^13–15^.

An alternative mechanism of replication fork plasticity is provided by the specialized primase PrimPol, mediating repriming of DNA synthesis in the face of obstacles^5,16^. This discontinuous mode of replication promotes bypass of bulky DNA lesions and implies generation of ssDNA gaps that are filled post-replicatively to complete genome duplication^17–21^. Fork reversal and repriming are competing options of DNA damage tolerance^5^, and fine-tuning their balance recently proved crucial to determine the response to genotoxic treatments^10,22^, and to enable proliferation bursts upon tissue-specific stimuli^23^. Recent evidence suggested that fork plasticity transactions are not limited to forks directly challenged by DNA lesions or replication interference, but rather rapidly extend to unchallenged forks as a global, nuclear response^24^. Although ATR - the central kinase of the human RS response - was implicated in this “signaling” mechanism controlling global fork progression and remodeling^24^, the underlying molecular mechanisms remain elusive and may involve nuclear architecture and dynamics^25,26^.

Actin is a well-characterized component of the cytoskeleton, involved in multiple cytoplasmic functions, mainly related to its ability to polymerize into filaments (F-actin). Although only a minority of actin resides in the nucleus, nuclear monomeric actin – along with several actin-binding proteins – is a stable component of several chromatin remodeling factors and RNA polymerases^27,28^. These findings provided possible explanations for the relevance of actin in DNA metabolism^29,30^. Filamentous actin structures on the other hand were long undetectable in the nucleus of most cell types. However, recent technological developments in actin filament detection^31^ – i.e. mainly the use of fluorescently labelled actin-binding domains fused to an NLS – allowed to reveal F-actin structures in the nucleus and linked them to various aspects of cell signaling, chromatin dynamics and DNA repair^28,29,32,33^. Dynamic and transient F-actin structures were reported in response to serum stimulation^34^ or upon integrin signaling during cell adhesion and spreading^35^. Nuclear F-actin structures are also induced upon T cell receptor (TCR) activation and nuclear accumulation of Ca^2+^, where they appear to control chromatin dynamics and transcription^36,37^. A role for nuclear F-actin in modulating chromatin condensation and nuclear volume was also reported upon mitotic exit^38^.

Importantly, recent evidence has linked nuclear F-actin to different aspects of genome maintenance. Nuclear F-actin structures with different morphologies were described in response to DNA damage^39^. *ARP2/3*-dependent long and dynamic nuclear actin filaments form in response to ionizing radiation and facilitate homologous recombination (HR) repair of heterochromatic double strand breaks (DSBs) in *Drosophila*^32,40^ and mouse cells^40^, through myosin-driven directed movement of repair sites to the nuclear periphery. Similarly, ARP2/3-mediated short actin structures cluster DSBs to favour their repair by HR in human cells^32,41,42^. Further, nuclear actin regulates DNA replication initiation upon S phase entry^43^ and was proposed to mediate mobility and repair of broken forks after prolonged fork stalling, via thick, long and persistent nuclear actin filaments^44^. A direct contact between actin nucleators and Replication Protein A (RPA) was recently reported to mediate DNA damage signaling and repair in both human and yeast cells^45^ and to limit nascent strand degradation at stalled forks^46^. Protection of stalled forks from nucleolytic degradation also requires the nuclear pool of the actin-based molecular motor myosin VI^44,47^. However, whether nuclear actin polymerization participates in the immediate response to mild replication interference, modulating fork progression and plasticity under permissive conditions for DNA synthesis remains elusive.

Here we show that transient nuclear actin filaments are detectable in unperturbed S phase, are rapidly increased by mild genotoxic treatments and are associated with replication factories. Impairing ARP2/3-mediated nuclear branched actin polymerization by chemical or genetic tools rapidly abolishes active replication fork slowing and remodeling into reversed forks. We report that defective nuclear actin polymerization leads to deregulated engagement of PrimPol at sites of DNA synthesis, which promotes fast and discontinuous DNA synthesis, impairs fork reversal and affects chromosome integrity upon genotoxic treatments. These findings establish transient nuclear F-actin structures as key players of the RS response, paving the way for mechanistic investigations on the role of these and other nuclear architecture components in controlling fork plasticity and the response to chemotherapeutic treatments.

## Results

### Distinct nuclear actin filaments form in unperturbed S phase and are induced by replication stress

To investigate how nuclear actin is organized in replicating cells and how it reacts to replication stress, we selected mild treatments with the topoisomerase I inhibitor camptothecin (CPT) or the topoisomerase II inhibitor etoposide (ETP), which were previously shown to significantly impact replication fork progression without detectable DSBs^11^, and which do not significantly alter nuclear actin levels (Supplementary Fig. 1a). We imaged U2OS cell lines stably expressing the nuclear actin marker NLS-actin-chromobody (nAC-GFP) and the replication fork marker PCNA chromobody (PCNA-CB-RFP) (Supplementary Fig. 1b). Using these cells and our imaging setup, we detected short actin structures rapidly induced by treatment with the calcium ionophore A23187^36^, as well as previously characterized nuclear actin filaments and patches forming upon mitotic exit^38^ (Supplementary Fig. 1c; Supplementary Movies 1 and 2). Intriguingly, unperturbed replicating (PCNA+) cells also occasionally display distinct actin filaments, which are longer and less abundant than those induced by mitotic exit and after calcium ionophore treatment in similar experimental conditions (Supplementary Fig. 1d, e; Supplementary Movie 3). Most of the structures are 1–5 μm long and remarkably transient (Supplementary Fig. 1i, j), frequently visible only for a single 20-s imaging timepoint (Supplementary Fig. 1e). Similar structures are detected with other tools for live imaging of nuclear F-actin, such as LifeAct or F-tractin (Supplementary Fig. 1f). ETP treatment induces the formation of new nuclear actin filaments, increasing their overall number in replicating cells, especially within the first imaging period after treatment (Supplementary Fig. 1e, g, h). Hence, distinct transient nuclear F-actin structures are detected in replicating cells and are rapidly induced by mild genotoxic treatments that affect replication fork progression. However, ETP-induced F-actin structures remain rare and transient events, with an average of one new filament forming per cell at any given time point (Supplementary Fig. 1g), or ~1.5 total filaments per cell at each time point (Supplementary Fig. 1h).

**Fig. 1:**
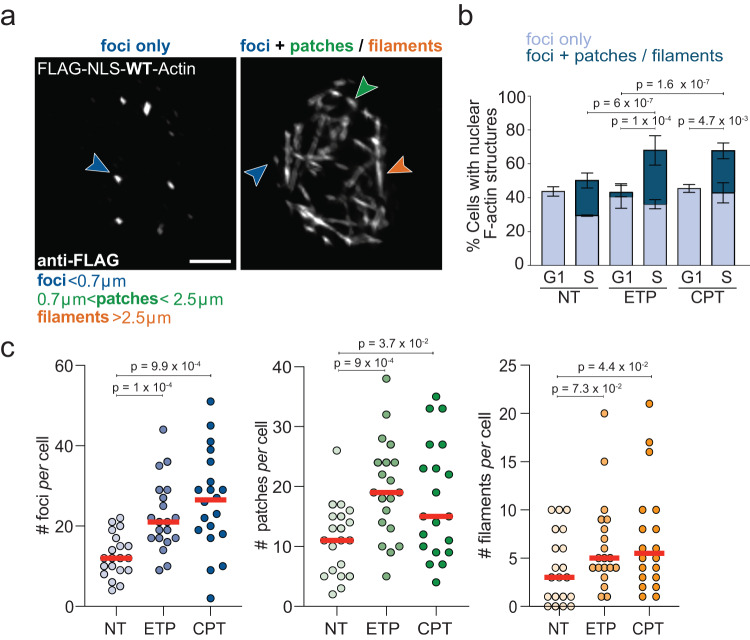
Nuclear F-actin structures in replicating cells upon mild genotoxic treatments. **a** Representative images of nuclear F-Actin structures detected in S phase U2OS cells, stably expressing FLAG-NLS-WT-Actin. Scale bar = 5 μm. According to their length, the structures were divided into three categories: foci (<0.7 μm), patches (>0.7 μm and <2.5 μm) and filaments (>2.5 μm). **b** Percentage of cells with actin structures (foci only or foci + patches/ filaments) in G1 (EdU-, diameter <15 μm based on DAPI) or S phase (EdU+). As indicated, cells were either left untreated (NT) or treated for 1 h with 20 nM etoposide (+ETP) or 100 nM camptothecin (+CPT). Data are mean ± SD; *N* = 404 (NT, S), *N* = 378 (ETP, S), *N* = 396 (CPT, S), *N* = 66 (NT, G1), *N* = 63 (ETP, G1), *N* = 53 (CPT, G1) from three independent experiments. Statistical analysis: two-tailed Mann–Whitney test applied to total F-actin structures. **c** Number of actin structures *per* cell. Cells were left untreated (NT) or treated for 1 h with 20 nM ETP or 100 nM CPT. Twenty cells were analyzed in each condition in three independent experiments. Red lines indicate the median. Statistical analysis: two-tailed Mann–Whitney test. See Supplementary Fig. 1p for compiled repetitions. Source data are provided as a Source Data file.

Although live-cell imaging by actin chromobody is a powerful tool to investigate the dynamics of nuclear F-actin^36,38,44^, it is intrinsically limited in its detection power by relatively low signal-to-noise ratio and typically detects only a subset of particularly visible structures^31,40,44^. Hence, to complement our observations with an alternative F-actin imaging tool and to increase signal-to-noise ratio, we investigated nuclear F-actin by immunofluorescence analysis of U2OS cells expressing FLAG-NLS-Actin^48,49^, where replication factories are identified by EdU incorporation. Fixed cell imaging of cells transiently transfected with this construct reveals a dense network of punctate and filamentous nuclear F-actin structures (Supplementary Fig. 1k). These structures are uniquely nuclear, as shown by middle-Z stack imaging of the nucleus with confocal microscopy (Supplementary Fig. 1k). Both punctate and filamentous structures correspond to polymerized actin, as they are largely lost in cells expressing the actin mutant R62D (FLAG-NLS-R62D-Actin), which acts by poisoning actin polymerization^38^ (Supplementary Fig. 1l). To avoid possible artifacts due to high expression levels of fluorescently tagged actin^29^ and to properly assess frequency, length and damage dependency of these F-actin structures, we isolated U2OS cells stably expressing particularly low levels of FLAG-NLS-Actin, which do not significantly alter total nuclear actin levels (Supplementary Fig. 1m, n), but allow nuclear F-actin visualization via FLAG staining of fixed cells (Fig. 1). In this controlled experimental system, we can detect defined punctate F-actin structures (“foci”; length <0.7 μm) in 30–40% of both G1 and S phase cells (Fig. 1a, b). Ca. 20% of S phase cells also display more elongated F-actin structures, which we classified as “patches” or “filaments” (Fig. 1a, b, Supplementary Fig. 1o). Importantly, a higher proportion of S phase cells forms F-actin patches or filaments in response to mild ETP or CPT treatments (Fig. 1b). The number of F-actin structures *per* replicating cell is also significantly and reproducibly increased upon both treatments, with foci and patches clearly representing the majority of the damage-induced structures (Fig. 1c, Supplementary Fig. 1o, p). Hence, specific F-actin structures are detectable in replicating cells and are rapidly induced upon mild genotoxic treatments known to impair replication fork progression.

### Nuclear actin filaments contact replication factories in an ARP2/3-dependent manner

**Fig. 2:**
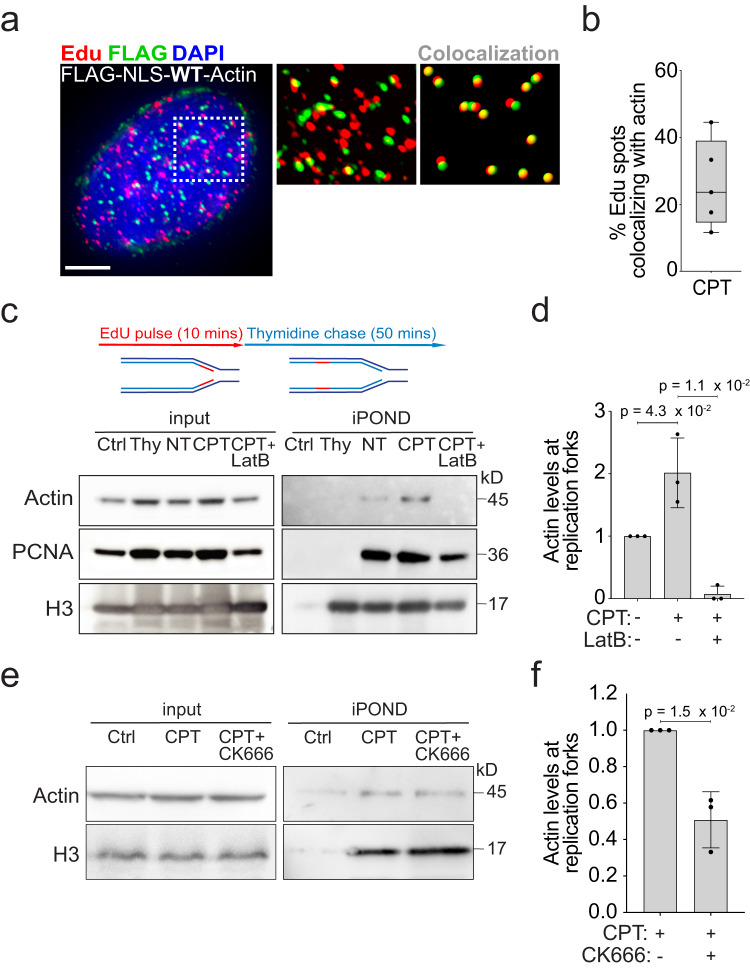
Nuclear F-actin interaction with replication factories and nascent DNA. **a** Representative image of a cell treated for 1 h with 100 nM CPT, stained for EdU and nuclear F-Actin (FLAG-NLS-Actin). Zoomed detail highlights EdU foci overlapping with F-Actin foci (yellow) quantified in Imaris. Scale bar = 10 μm **b** Quantification of Fig. 2a (see “Methods” for details) performed on 5 cells displayed as black dots. Box extends from the 25th to the 75th percentile. Line in the box represents the median. Whiskers extend down to the smallest and up to the highest value. **c–f** Isolation of proteins on nascent DNA (iPOND). **c** Top: Simplified experimental setup: 10 min EdU pulse in HEK293T cells optionally followed by a 50 min thymidine chase (Thy), discriminating chromatin-associated proteins behind replication forks. See Supplementary Fig. 2a for details. Bottom: Western blot analysis of cells optionally treated with CPT (100 nM, 1 h). Where indicated, LatB (100 nM) was added 10 min prior to CPT and retained. Proteins associated with nascent DNA were isolated by iPOND and detected with the indicated antibodies. Click reaction is performed using DMSO instead of biotin azide as a specificity control (Ctrl). **d** Graph-bar depicts mean ± SD of Actin protein levels at nascent DNA from three independent experiments (black dots). Values are normalized to H3 and displayed as fold change over NT. Statistical analysis: One-tailed t-test with Welch’s correction. **e** Western blot analysis of cells optionally treated with CPT (100 nM, 1 h). Where indicated, 100 nM CK666 was added 10 min prior to CPT and retained. Proteins associated with nascent DNA were isolated by iPOND and detected with the indicated antibodies. Ctrl sample as in (**c**). **f** Graph-bar depicts mean ± SD of Actin protein levels at nascent DNA from three independent experiments (black dots). Values are normalized to H3 and displayed as fold change over CPT. Statistical analysis: one-tailed *t*-test with Welch’s correction. Source data are provided as a Source Data file.

To assess the possible physical proximity between F-actin structures and replication factories, we selected a few CPT-treated S phase (EdU+) cells displaying a clear punctate F-actin pattern. Quantification of these signals shows that 20–40% of replication factories (EdU foci) overlap with F-actin foci (Fig. 2a, b). To further assess F-actin proximity to DNA replication centers and estimate how this is affected by mild genotoxic treatments, we took advantage of *isolation of proteins on nascent DNA* (iPOND^50^) (Fig. 2c, d; Supplementary Fig. 2a). Immunoprecipitation of nascent DNA identifies actin at DNA synthesis centers, confirming that actin is detected proximal to replication factories in unperturbed S phase (Fig. 2c). Remarkably, this interaction is significantly enhanced upon CPT treatment and markedly reduced when the actin polymerization inhibitor LatrunculinB (LatB) is added to the media shortly (10 min) before CPT (Fig. 2c, d; Supplementary Fig. 2a). Although actin polymerization can be promoted by various co-factors, branched actin filament formation uniquely requires the ARP2/3 complex, which can be specifically inhibited by CK666 treatment^51^. A 10-min preincubation of cells with CK666 prior to CPT treatment mildly but significantly reduces the interaction of F-actin with nascent DNA, suggesting that the branched actin network represents at least a portion of polymerized nuclear actin upon damage (Fig. 2e, f). Given the previous evidence that nuclear F-actin structures promote the dynamic repositioning of broken forks, we assessed the movement of replication centers in our conditions. 3D tracking of PCNA foci and mean square displacement (MSD) analysis shows that ETP treatment does not affect the dynamics of bulk replication factories (Supplementary Fig. 2b), consistent with most forks not being broken in these conditions^11^. Hence, upon mild genotoxic treatments, nuclear actin polymerization takes place in proximity to a significant fraction of replication factories, but does not detectably affect their mobility.

### ARP2/3-dependent nuclear actin polymerization is required for active fork slowing upon replication stress

**Fig. 3:**
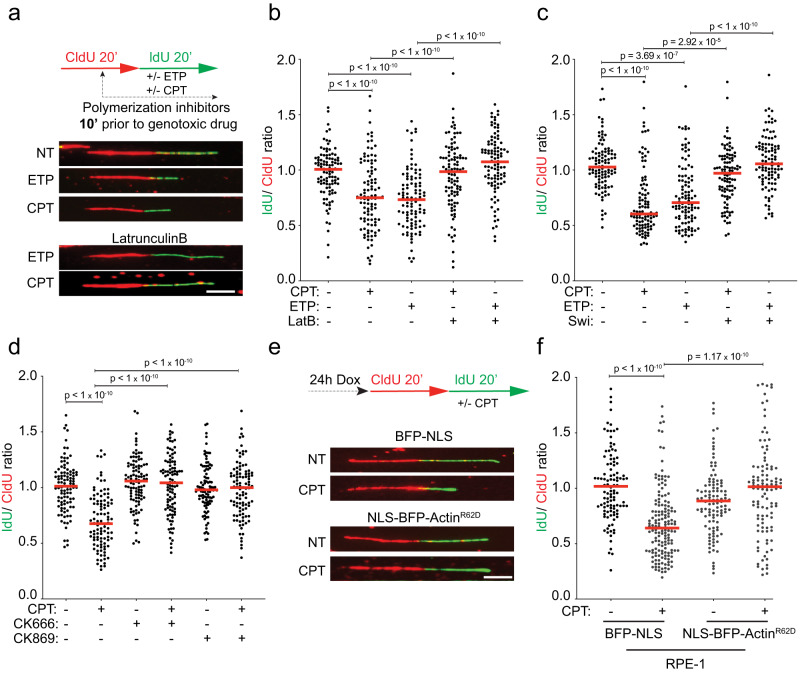
Nuclear actin polymerization is required for active fork slowing upon mild genotoxic stress. **a**–**d** DNA fiber analysis of U2OS cells. **a** Top: Schematic CldU/IdU pulse-labeling protocol used to evaluate fork progression upon 100 nM CPT or 20 nM ETP. 100 nM LatB, Swi, CK666 or CK869 were added 10 min prior to CPT or ETP and retained during the IdU labelling. Bottom: Representative DNA fiber images. Scale bar = 5 μm. **b**–**d**, **f** IdU/CIdU ratio is plotted for a minimum of 100 forks (indicated as black dots) from a single representative experiment. Red line indicates the median. See Supplementary Fig. 3b–d, h for compiled repetitions (*n* = 3). Statistical analysis: two-tailed Mann–Whitney test. **e**, **f** DNA fiber analysis of RPE-1 cells stably expressing doxycycline inducible BFP-NLS or NLS-BFP-Actin^R62D^. **e** Top: schematic of the CldU/IdU pulse-labeling protocol used to evaluate fork progression upon 100 nM CPT. Doxyclicline (Dox) was added 24 h before CldU/IdU pulse-labeling. Bottom: representative DNA fibers images. Scale bar = 5 μm. Source data are provided as a Source Data file.

We next investigated the functional relevance of the interaction between nuclear F-actin and replication factories on DNA replication, in presence or absence of mild genotoxic treatments. To this purpose, we analyzed replication fork progression at single-molecule level by spread DNA fiber assays^52^, providing cells with halogenated nucleotides and mild doses of ETP or CPT. These treatments were previously shown to induce marked fork slowing and reversal, with no major impact on cell cycle progression, chromosome integrity and cell viability^11^. In this setup, we induced actin depolymerization - adding LatB or Swinholide A (Swi), an alternative actin depolymerization agent - when incorporation of halogenated nucleotides was already ongoing, i.e. 10 min before addition of the genotoxic drugs (Fig. 3a). Pre-treatment with LatB or Swi does not detectably affect replication fork progression (Supplementary Fig. 3a), showing that actin polymerization is per se not required to support efficient fork progression in unperturbed conditions. As expected from previous studies, both ETP and CPT drastically affect replication fork progression, but the active fork slowing observed in these conditions is significantly rescued by either of the actin polymerization inhibitors (Fig. 3a–c and Supplementary Fig. 3b, c). Moreover, a very similar effect of LatB is observed when treating cells with a different agent inducing DNA lesions (cisplatin), instead of a topoisomerase inhibitor (Supplementary Fig. 3d)^53^. Similarly to LatB or Swi, 10 min pretreatment with either CK666 or the alternative ARP2/3-inhibitor CK869^51^ has no impact on unperturbed DNA synthesis per se (Supplementary Fig. 3a), but abolishes CPT-induced fork slowing (Fig. 3d and Supplementary Fig. 3e). Moreover, siRNA-mediated downregulation of *ARP3* has very similar effects (Supplementary Fig. 3f, g), suggesting that the branched actin network plays a pivotal role in modulating replication fork progression upon DNA damage.

Although the minimized timing of treatment excludes long-term pleiotropic effects, chemical inhibition of actin polymerization may unavoidably affect the bulk actin network in the cytoplasm. To specifically investigate the functional relevance of nuclear F-actin in the modulation of replication fork progression, we took advantage of a previously established genetic tool, i.e. a stable RPE-1 cell line bearing a doxycycline-inducible R62D actin mutant (NLS-BFP-Actin^R62D^)^35,38,48^. Due to the NLS, this exogenous actin specifically impairs nuclear actin polymerization, but does not detectably affect cytoplasmic F-actin and its functions^38,40^. Remarkably, induction of NLS-Actin^R62D^ 24 h before our fiber assays also fully suppresses CPT-induced fork slowing (Fig. 3e, f and Supplementary Fig. 3h), establishing nuclear F-actin assembly as key molecular determinant of the rapid modulation of replication fork progression upon mild genotoxic treatments.

### Nuclear actin polymerization mediates the engagement of fork remodelling factors and fork reversal

**Fig. 4:**
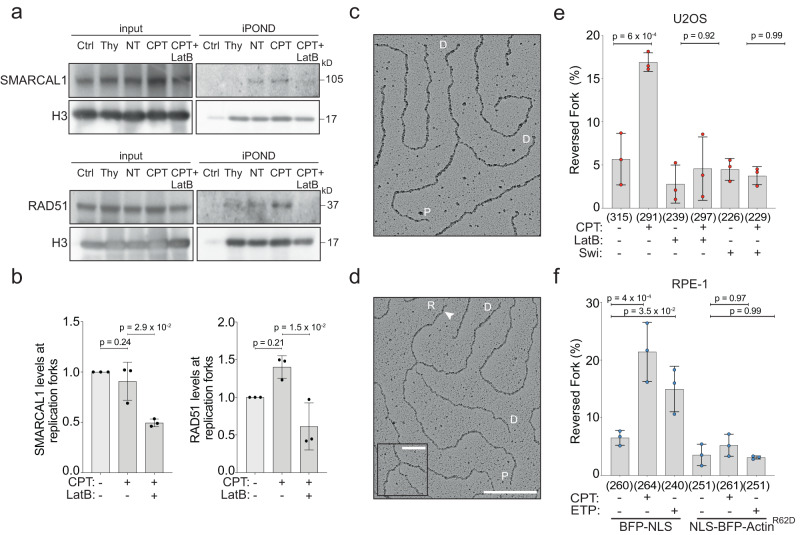
Nuclear F-actin modulates the engagement of replication fork remodellers and fork reversal. **a** iPOND analysis of HEK293T cells after indicated treatments (100 nM CPT, 1 h; 100 nM LatB, 10 min prior to CPT). See Supplementary Fig. 2a for details. Proteins associated with nascent DNA were isolated by iPOND and detected with the indicated antibodies. In the control (Ctrl) experiment, the click reaction is performed using DMSO instead of biotin azide. Same representative experiment as in Fig. 2c, d. **b** Graph-bar depicts mean and SD of quantified RAD51 and SMARCAL1 levels at nascent DNA from three independent iPOND experiments (black dots). Values are normalized to H3 and represented as fold change over the NT sample. Statistical analysis: one-tailed t-test with Welch’s correction. **c**, **d** Electron micrographs of representative replication forks from U2OS cells: parental (P) and daughter (D) duplexes. **d** White arrow indicates the regressed arm (R); the four-way junction at the reversed fork is in the inset. Scale bar = 200 nm, 40 nm in the inset. **e** Frequency of reversed replication forks isolated from U2OS cells upon optional treatment with 100 nM CPT for 1 h. 100 nM LatB or Swi were added 10 min before CPT and retained during the genotoxic treatment. Total number of molecules analyzed per condition in brackets. **f** Frequency of reversed replication forks isolated from RPE-1 cells after 24 h doxycycline-induction of either BFP-NLS or NLS-BFP-Actin^R62D^ and optional treatment with CPT (100 nM, 1 h) or ETP (20 nM, 1 h). Total number of molecules analyzed per condition in brackets. **e**, **f** Bar graphs depict mean ± SD from three independent EM experiments (red and blue dots, respectively). Statistical analysis: ordinary one-way ANOVA. Source data are provided as a Source Data file.

Active replication fork slowing upon DNA damage or mild replication interference was linked to replication fork reversal, i.e. the controlled and reversible remodeling of replication forks into four-way junctions^5^. This transaction requires the recruitment to forks of the RAD51 recombinase^11,12^ and active engagement of the specialized translocase SMARCAL1, which is a stable component of the replisome^8,54^. We thus used the iPOND approach described in Fig. 2 and Supplementary Fig. 2 to investigate whether nuclear F-actin is required to enable recruitment or engagement of these remodeling factors on nascent DNA. In agreement with previous observations^8^, the presence of RAD51 on nascent DNA is significantly increased upon CPT treatment, while SMARCAL1 is detectable at replication forks at comparable levels in treated and untreated cells (Fig. 4a, b). Remarkably, treating cells with LatB 10 min prior to CPT – which was shown in Fig. 2 to markedly suppress actin proximity to replication forks – also reduces the levels of both RAD51 and SMARCAL1 on nascent DNA (Fig. 4a, b). These data suggest that blocking actin polymerization affects the recruitment to replication forks or the engagement of these factors at sites of DNA synthesis, which may prevent their efficient crosslinking and detection on nascent DNA.

We then directly assessed whether defective active polymerization affects the ability of the cells to promptly induce replication fork reversal upon genotoxic stress. For this purpose, we took advantage of an established approach for direct electron microscopic visualization of replication intermediates^53,55^, which allows distinguishing standard 3-way replication forks from 4-way reversed forks (Fig. 4c, d). In line with previously published data^11^, both U2OS and RPE-1 cells display a drastic and reproducible increase in the percentage of reversed forks upon CPT or ETP treatment (Fig. 4e, f and Supplementary Fig. 4a, b). Remarkably, treatment with either LatB or Swi 10 min before CPT treatment fully abolishes drug-induced replication fork reversal in U2OS cells (Fig. 4e and Supplementary Fig. 4a). Moreover, expression of the NLS-Actin^R62D^ dominant-negative mutant also impairs CPT- and ETP-induced reversal in RPE-1 cells (Fig. 4f and Supplementary Fig. 4b), establishing nuclear actin polymerization as a strict requirement for active fork slowing and reversal in human cells.

Previous work had linked the central kinase of the replication stress response ATR with efficient replication fork reversal^24^. Moreover, nuclear F-actin assembly was recently suggested to mediate local ATR activation upon laser micro-irradiation^56^. However, neither chemical nor genetic inactivation of nuclear actin polymerization resulted in a detectable impairment of the phosphorylation of the key ATR target CHK1 (Supplementary Fig. 4c, d), suggesting that the role of nuclear F-actin in replication fork progression and remodeling is independent or downstream from canonical ATR activation.

### Nuclear actin polymerization promotes fork remodeling by limiting PrimPol-mediated repriming

**Fig. 5:**
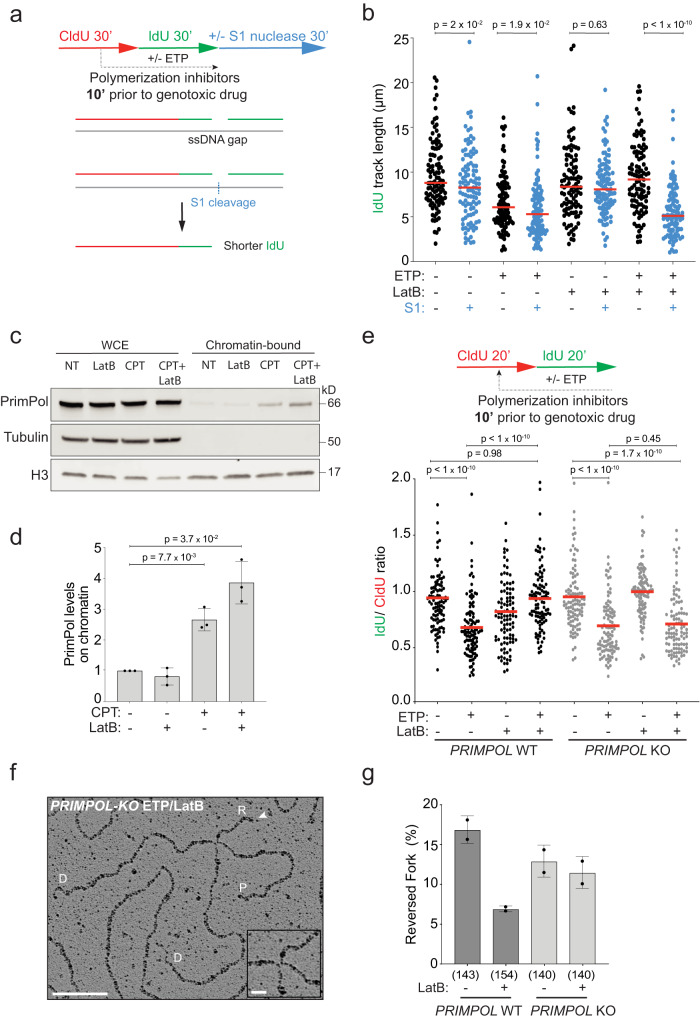
PrimPol deregulation prevents efficient fork remodeling upon defective actin polymerization. **a** Timeline of CldU/IdU pulse-labeling coupled with 30 min S1 nuclease treatment to detect ssDNA gaps on nascent DNA upon optional treatment with 20 nM ETP. 100 nM LatB was added 10 min prior to ETP and retained. **b** IdU track length (μm) is plotted as readout of discontinuous DNA synthesis for a minimum of 100 forks (black or blue dots) per sample in a single, representative experiment. Red lines indicate the median. See Supplementary Fig. 5d for compiled repetitions (*n* = 3). Statistical analysis: two-tailed Mann–Whitney test. **c** Representative immunoblot of the indicated proteins in whole cell extracts (WCE) or the chromatin bound fraction. **d** Bar graph depicts mean ± SD of chromatin bound PrimPol levels from three independent experiments from c (black dots). Values are normalized to H3 and represented as fold change over NT. Statistical analysis: one-tailed t-test with Welch’s correction. **e** DNA fiber analysis of U2OS *PRIMPOL* WT and KO cells. Top: CldU/IdU pulse-labeling protocol to evaluate fork progression upon 20 nM ETP. 100 nM LatB was added 10 min prior to ETP and retained during the IdU labelling. Bottom: IdU/CldU ratio plotted for a minimum of 100 forks per sample (black dots) from a single, representative experiment. Red line indicates median. See Supplementary Fig. 5e for compiled repetitions (*n* = 3). Statistical analysis: two-tailed Mann–Whitney test. **f** Representative electron micrograph of a reversed fork isolated from *PRIMPOL* KO cells, priorly treated with ETP and LatB; parental (P) and daughter (D) duplexes. White arrow indicates the regressed arm (R); the four-way junction at the reversed fork is magnified in the inset. Scale bar = 100 nm, 10 nm in the inset. **g** Frequency of reversed replication forks isolated from U2OS cells (proficient (WT) or deficient (KO) for PRIMPOL) upon 1 h of 20 nM ETP. 100 nM LatB was added where indicated 10 min before ETP and retained. Bar graph depicts mean ± SD from two independent EM experiments (black dots). Total number of molecules analyzed per condition in brackets. Source data are provided as a Source Data file.

We next investigated the genetic dependencies of the unrestrained fork progression observed upon replication interference when nuclear actin polymerization is impaired. Accelerated DNA synthesis was previously reported upon PARP inhibition and was linked to deregulated fork restart activity of the RECQ1 helicase, which prevents fork pausing in the reversed state^11,57,58^. However, effective downregulation of *RECQ1* by siRNA does not restore active fork slowing upon ETP treatment, in the presence of LatB (Supplementary Fig. 5a–c). Thus, unrestrained fork progression upon defective nuclear actin polymerization does not reflect accelerated restart of previously reversed forks. We next assessed whether defective fork slowing was linked to deregulated de novo restart of DNA synthesis on a damaged template, which was recently reported in other genetic conditions impairing fork reversal^10,22,59^. This discontinuous mode of DNA synthesis implies the transient formation of ssDNA gaps on newly replicated duplexes and can be detected in a modified DNA fiber assay as shortening of replicated tracks by cleavage of the ssDNA-specific S1 nuclease, prior to fiber stretching on microscopy slides^60^ (Fig. 5a). Indeed, S1-induced replication track shortening is specifically detected in our assays when ETP is combined with LatB pre-treatment (Fig. 5b and Supplementary Fig. 5d), confirming that unrestrained fork progression upon defective nuclear actin polymerization entails discontinuous DNA synthesis on a damaged template.

This discontinuous DNA synthesis has been previously linked to the action of the PrimPol primase^10,16–19,22^; thus, we investigated PrimPol engagement in DNA synthesis in our experimental conditions. To do so, we relied on chromatin fractionation and indeed observed that PrimPol chromatin loading is induced by CPT treatment – as previously reported upon induction of DNA damage^17,20,59^ – and further enhanced by pre-treatment with LatB (Fig. 5c, d). We next directly assessed the genetic contribution of *PRIMPOL* performing DNA fiber assays on PRIMPOL-KO U2OS cells and confirmed that the unrestrained fork progression induced by LatB in the presence of ETP is indeed entirely dependent on *PRIMPOL* (Fig. 5e and Supplementary Fig. 5e). Importantly, *PRIMPOL* dependency for unrestrained fork progression is also observed when ARP2/3-dependent branched nuclear F-actin is impaired by CK666 treatment (Supplementary Fig. 5f–g). Hence, defective nuclear actin polymerization rapidly provides deregulated access of PrimPol to damaged replication forks, promoting excessive repriming and discontinuous DNA synthesis. A deregulated balance between fork reversal and repriming was previously shown upon various genetic conditions affecting one or the other mechanism^10,22,61,62^. However, *PRIMPOL* inactivation restored slow fork progression in ETP despite defective nuclear actin polymerization (Fig. 5e), suggesting that deregulated PrimPol may represent the primary defect, leading to impaired fork reversal in these conditions. To directly test this hypothesis, we performed EM experiments in *PRIMPOL*-KO U2OS cells; despite a minor effect of *PRIMPOL* inactivation on the frequency of reversed forks in ETP treated cells, we found that *PRIMPOL* defective cells do not experience the ca. threefold drop in fork reversal frequency induced in control cells by LatB treatment (Fig. 5f, g and Supplementary Fig. 5h). Hence, upon *PRIMPOL* inactivation, cells are capable to promote fork reversal even in the absence of nuclear F-actin.

### Nuclear actin polymerization limits genomic instability upon DNA damage by regulating PrimPol

**Fig. 6:**
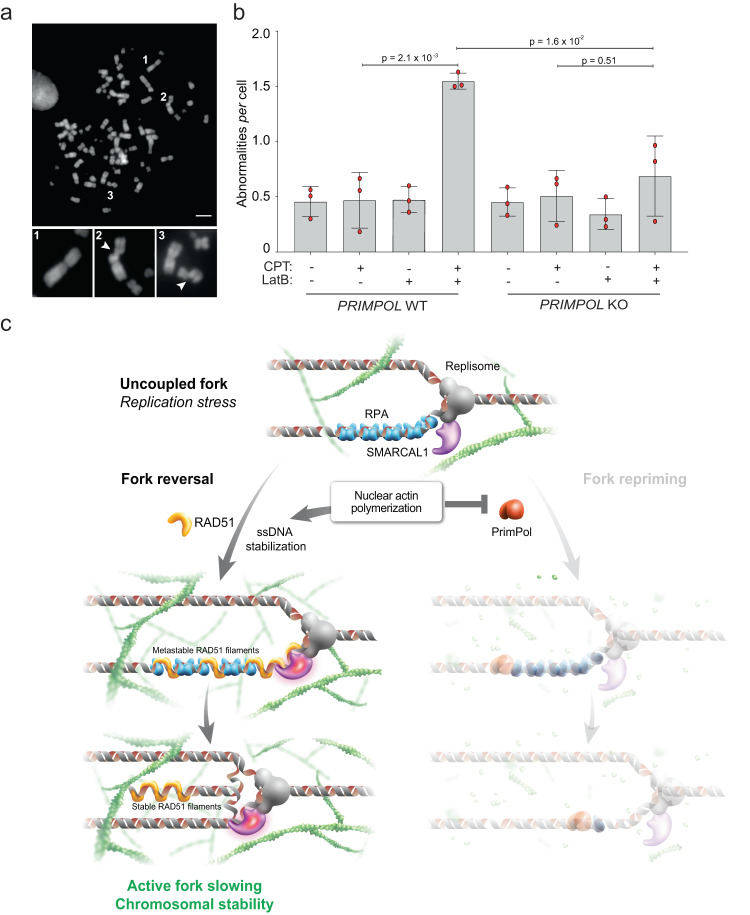
Nuclear F-actin limits chromosomal instability upon genotoxic treatments by limiting PrimPol function and promoting efficient fork remodeling. **a** Representative metaphase spread image. Scale-bar = 5 μm. 1 = representative intact chromosome. 2, 3 = representative breaks. **b** Number of chromosomal abnormalities in U2OS cells (proficient for (WT) or lacking (KO) *PRIMPOL*) optionally treated with 100 nM CPT for 2 h. Where indicated, 100 nM LatB was added 10 min before CPT and retained. Bar graph depicts mean ± SD from three independent experiments (red dots). A minimum of 30 metaphases was analyzed per sample and experiment. Statistical analysis: one-way ordinary ANOVA. **c** Working model: nuclear F-actin polymerization limits PrimPol recruitment to uncoupled replication forks, stabilizing ssDNA stretches. This facilitates RAD51 recruitment to stalled/uncoupled forks and promotes SMARCAL1-dependent fork reversal, mediating active fork slowing and protecting the integrity of replicating chromosomes. A graphical representation of the consequences of defective nuclear actin polymerization is depicted in Supplementary Fig. 6c (see Discussion for details). Source data are provided as a Source Data file.

Finally, we investigated the functional consequences of nuclear F-actin deregulation on the cellular response to replication stress, in terms of chromosome stability. We used chromosome spreads from metaphase arrested cells to monitor chromosomal breaks and abnormalities (Fig. 6a). Using mild CPT treatments that do not induce per se significant chromosomal instability in U2OS cells, we observe a marked increase of chromosomal instability when actin polymerization is impaired by LatB shortly before CPT treatment (Supplementary Fig. 6a). Similarly, specific impairment of nuclear F-actin by inducible expression of NLS-Actin^R62D^ in RPE-1 cells increases CPT-induced chromosomal abnormalities (Supplementary Fig. 6b). Strikingly, PRIMPOL inactivation completely suppresses the genomic instability induced by defective nuclear actin polymerization in CPT-treated cells, clearly linking the observed chromosomal instability to defective replication fork plasticity and in particular to deregulated PrimPol activity.

## Discussion

Our data identify a novel role of nuclear F-actin structures in DNA replication in human cells. We provide several lines of evidence that nuclear F-actin plays a pivotal role in orchestrating the rapid response to replication interference, promoting chromosome stability upon mild genotoxic treatments. Specifically, we detect transient and dynamic F-actin structures that form in a normal S phase, mostly represented by small foci and patches, and more rarely by longer filaments. All of these structures are rapidly induced by replication stress and are associated with replication sites. F-actin structures observed in this context are distinct from filaments and patches previously detected by actin-CB expression in response to various stimuli^34,35,37,38^ – including prolonged fork stalling^44^ – and may largely escape detection by standard live imaging given their thin structure and transient nature. This was recently exemplified by the identification of novel and transient “actin droplets” mediating androgen signaling, which could only be detected by super-resolution live-cell microscopy^63^. Although the use of these refined imaging approaches in future studies will likely reveal additional mechanistic details, the fixed cell imaging we performed by spiking endogenous nuclear actin with low amounts of tagged actin already allowed us to reveal a complex network of F-actin structures, establishing frequent contacts with replication sites and extending upon replication stress, consistent with a global role of F-actin in replication fork plasticity.

We show that nuclear actin polymerization acts as a critical determinant in the choice between alternative mechanisms of DNA damage tolerance during replication, i.e. replication fork reversal vs repriming. Our data suggest that nuclear F-actin is per se dispensable for fork reversal; although we cannot exclude that nuclear actin polymerization may also facilitate fork remodelling, our data strongly suggest the regulation of PrimPol as key molecular function of nuclear actin polymerization in orchestrating fork plasticity and protecting chromosome integrity upon mild replication interference. Both, fork reversal and repriming require accumulation of RPA-coated ssDNA^11,17^, which is typically observed upon stalling of leading strand synthesis at DNA lesions, while unwinding by the replicative helicase and lagging strand synthesis proceed beyond the lesion (uncoupled fork; Fig. 6c). However, while PrimPol-mediated repriming is promoted by direct interaction with RPA and de novo DNA synthesis by its primase activity^64^, replication fork reversal is a complex reaction^5^, requiring partial exchange of RPA with RAD51 – catalysed by the action of RAD51 paralogs^11,65^ – and the concerted action of SMARCAL1, ZRANB3 and HLTF translocases^8–10^, differentially activated by RAD51 and its cofactors^54^ (Fig. 6c). We propose that polymerizing F-actin in proximity to replication factories may limit deregulated access of PrimPol to ssDNA, which would lead to an excessively discontinuous DNA synthesis and may saturate the cellular capacity for post-replicative gap-filling^19,21^. Considering that RPA-coated ssDNA is proposed to extrude as a loop from the replication center, preventing unlimited access of PrimPol to RPA-coated ssDNA may be required to stabilize the uncoupled fork and to kinetically allow the complex biochemical reactions required for fork reversal. In this context, defective nuclear actin polymerization could provide deregulated access of PrimPol to ssDNA gaps at uncoupled forks, rapidly filling ssDNA regions and thereby preventing fork remodeling (Supplementary Fig. 6c). The impact of PrimPol deregulation on chromosomal instability upon mild genotoxic stress is in line with recent evidence: although PrimPol activation may promote cellular resistance to genotoxic treatments in specific genetic backgrounds that are defective for replication fork remodelling or protection^10,22^, its excessive activation was also recently linked to reduced cellular fitness and increased chromosomal instability^61,62^, highlighting the physiological relevance of keeping PrimPol activity under tight control.

How nuclear F-actin specifically controls PrimPol activity and whether this response entails signaling pathways previously involved in the replication stress response is currently unknown. However, we propose here several hypotheses on how nuclear actin polymerization limits PrimPol access, rapidly and globally affecting replication fork plasticity upon genotoxic treatments. Similarly to what has been described upon mitotic exit^38^, nuclear F-actin may be required upon RS to locally modify chromatin compaction. However, differently from the global chromatin decondensation observed during nuclear volume expansion in G1 cells, replicating cells may use the nuclear F-actin network to locally and transiently increase chromatin compaction, which could help executing the replication program and responding rapidly to RS. Intriguingly, changes in chromatin compaction and epigenetic marks were reported upon multiple sources of RS and proposed to mediate nuclear positioning of forks experiencing prolonged stalling^26^. Most recently, heterochromatic marks were shown to rapidly accumulate on nascent DNA upon fork stalling, modulating the recruitment of accessory replication factors that mediate replication fork protection and restart^66^. We propose that local actin polymerization in proximity to replication forks is part of this emerging mechanism that limits access of distributive and potentially dangerous replication factors (e.g. PrimPol) and ensures replication fork plasticity and genome integrity upon replication stress. Fine-tuning PrimPol activity seems crucial for cellular fitness and drug response, as enhanced repriming provides chemoresistance in specific genetic backgrounds^10,22^, but can also overall increase chromosomal instability^61,62,66^. Unraveling structures and molecular mechanisms mediating PrimPol control by nuclear F-actin will be a fascinating challenge for future studies.

Nuclear F-actin could also affect fork plasticity by modifying the nuclear position or dynamics of replication sites. Differently from DSB repair^32,40,42^ and prolonged fork stalling or collapse^44^, we did not detect relocation of bulk replication factories within the experimental time frame upon mild RS, suggesting that extensive relocalization is not needed to limit PrimPol engagement and modulate fork plasticity^44^. It is however possible that short-range movement of bulk replication sites – hardly detectable with current imaging resolution – contributes to orchestrate replication fork plasticity. Although limiting PrimPol access appears as the central function of nuclear F-actin in the immediate replication stress response, it is also possible that complex biochemical reactions – such as extensive DNA unwinding and annealing driving fork reversal – may require increased short-range mobility of nascent DNA mediated by local nuclear actin polymerization, analogously to the sudden changes in chromosome dynamics that promote homologous pairing in meiosis^67^. As chromosome mobility and directed movement is linked to efficient DNA repair^40,42,68–73^, it will be essential to thoroughly investigate the functional relevance of myosin and other motor proteins in the local, actin-mediated modulation of replication fork plasticity, especially in light of the recently reported role of myosin VI in stalled fork protection^47^. Finally, although our data strongly suggest a direct involvement of F- actin in regulating fork plasticity at replication factories, they do not exclude that local changes in monomeric actin linked to its polymerization may participate in these processes, possibly via its direct interaction with RPA^46^ or via its participation in chromatin remodeling complexes^74^.

Further investigation will be needed to test these and alternative hypotheses, to uncover detailed mechanisms and signaling pathways mediating the role of nuclear F-actin in the replication stress response. Regardless of the molecular mechanisms, the surprising impact of nuclear F-actin on chromosome integrity upon mild replication interference suggests that specific players involved in nuclear actin polymerization may affect the response to cancer chemotherapy, highlighting the clinical relevance of further mechanistic investigations in this area.

## Methods

### Key materials

All antibodies and specific chemicals used for this study are available as tables in Supplementary Data 1 and 2, respectively. The experimental conditions to use them in the corresponding assays can be found in the dedicated method sections.

### Cell lines and plasmids

Human osteosarcoma U2OS cells, retinal pigment epithelium RPE-1 cells and HEK293T cells were cultured in DMEM (41966-029, Life Technologies) supplemented with 10% fetal bovine serum (FBS, GIBCO), 100 U/ml penicillin and 100 mg/ml streptomycin in an atmosphere containing 6% CO_2_ at 37 °C. *PRIMPOL KO* and isogenic U2OS cells were kindly provided by Dr. Juan Méndez. Nuclear-actin-chromobody (nAC-GFP) stable U2OS cells and stable doxycycline-inducible BFP-NLS or NLS-BFP-Actin^R62D^ cells were kindly provided by Dr. Robert Grosse. pEF-Flag-NLS-Actin-WT and R62D plasmids were kindly provided by Dr. Guido Posern^49^. LifeAct-GFP-NLS or F-tractin-GFP-NLS plasmids were kindly provided by Dr. Robert Grosse. The PCNA-chromobody (PCNA-CB-RFP) was transiently transfected in nAC-GFP stable U2OS cells with Lipofectamine 3000 (ThermoFisher Scientific) according to the manufacturer’s instructions and 24 h before imaging. U2OS cells were similarly transiently transfected with FLAG-NLS-WT or FLAG-NLS-R62D-Actin.

For the generation of U2OS-FNA cells, FLAG-NLS-Actin-WT was subcloned into a lentiviral expression plasmid (pLL5.0) and transfected with packaging vectors into HEK293T. Virus-like particles were harvested after 48 h and U2OS cells were transduced for 24 h. Afterwards, single clones were picked, grown, and tested for FLAG-NLS-Actin expression.

### RNAi experiments

For RNAi experiments, U2OS cells were transfected with the indicated siRNAs for 48 h: siLuc (5′-CGUACGCGGAAUACUUCGAUUdTdT-3′) and siRECQ1 (5’-UUACCAGUUACCAGCAUUAdTdT-3’); using jetPRIME (Polyplus transfection) according to manufacturer’s instruction. siLuc (5′-CGUACGCGGAAUACUUCGAUUdTdT-3′) and siARP3 (SMARTpool siRNA L-012077-00-0010 (Dharmacon)) were transfected using RNAiMax (Thermo Fisher Scientific) according to manufacturer’s instruction.

### Biochemical fractionation, protein extraction and western blotting

Biochemical fractionation was performed as described^20^ with minor modifications:

Cells were resuspended (4 × 10^7^ cells/ml) in buffer A (10 mM HEPES (pH 7.9), 10 mM KCl, 1.5 mM MgCl_2_, 0.34 M sucrose, 10% glycerol, 1 mM DTT, 1X complete Protease inhibitors cocktail (Roche)). Triton X-100 (0.1%) was added and cells were incubated for 5 min on ice. Nuclei were collected by low-speed centrifugation (4 min, 1300 × *g*, 4 °C). The supernatant (cytoplasmic fraction) was further clarified by high-speed centrifugation (15 min, 20,000 × *g*, 4 °C) to remove cell debris and insoluble aggregates. Nuclei were washed once in buffer A (nuclei fraction). To isolate chromatin, nuclei were further lysed in buffer B (3 mM EDTA, 0.2 mM EGTA, 1 mM DTT, protease inhibitors as described above). Insoluble chromatin was collected by centrifugation (4 min, 1700 × *g*, 4 °C), washed once in buffer B, and centrifuged again under the same conditions. The final chromatin pellet (chromatin fraction) was resuspended in Laemmli buffer, sonicated and loaded for conventional western blot.

Whole cell extracts or cellular fractions from all cell lines were prepared in Laemmli sample buffer. For cell extracts, equal amounts of protein (30–50 μg) were loaded onto 4–20% Mini-PROTEAN TGX Precast Protein Gels (BioRad). Proteins were separated by electrophoresis at 16 mA followed by transferring the proteins to Immobilon-P membranes (Thermo Fisher Scientific) for 1 h at 350 mA (4 °C) in transfer buffer (25 mM Tris and 192 mM glycine) containing 10% methanol. Before addition of primary antibodies, membranes were blocked in 5% milk in 0.1% TBST (1 × TBS supplemented with 0.1% Tween 20) for 1 h and incubated in 3% BSA with primary antibodies overnight at 4 °C. Membranes were probed for PrimPol (neat tissue culture supernatant, Méndez lab^20^), Tubulin (1:10,000, T5178, Sigma-Aldrich); H3 (1:5000, ab1791, Abcam); Arp3 (1:500, sc-48344, Santa Cruz Biotechnology); TFIIH (1:2000, sc-293, Santa Cruz Biotechnology); pChk1 (1:500, 2348, Cell Signaling Technology); Chk1 (1:1000, sc-8408, Santa Cruz Biotechnology); Recq1 (1:2000, Novus Biologicals, NB100-618); Gapdh (1:10,000, MAB374, Millipore). Membranes were washed three times with TBS/0.1% Tween-20 and incubated with secondary antibody (anti-mouse-HRP or anti-rabbit-HRP 1:5000 in 5% blocking solution) at RT for 1 h. Membranes were washed three times with 0.1% TBST, 10 min each, after primary and secondary antibody incubations and detected with ECL detection reagent (GE healthcare).

### Replication fork progression by DNA fiber analysis

All cell lines subjected to this analysis were grown asynchronously and labeled with 30 μM of the thymidine analog chlorodeoxyuridine (CldU; Sigma-Aldrich) for 20–30 min (as indicated), they were then washed three times with warm PBS and subsequently exposed to 250 μM of 5-iodo-2′-deoxyuridine (IdU) for an equal amount of time alone or in combination with mild doses of genotoxic treatments (100 nM CPT, 20 nM ETP, 20 μM Cisplatin). To evaluate the impact of actin polymerization on replication fork progression, actin polymerization inhibitors were added 10 min before the genotoxic treatment and retained during the IdU labeling. In the RPE-1 cells, 1 μM/mL Dox was added 24 h before the CldU/IdU pulse labeling. All cells were collected by standard trypsinization and resuspended in cold PBS at 3.5 × 10^5^ cells/mL. Three microliters of this cell suspension was then mixed with 7 μL of lysis buffer (200 mM Tris-HCl, pH 7.5, 50 mM EDTA, and 0.5% [w/vol] SDS) on a glass slide. After an incubation of 9 min at RT, the slides were tilted at a 45° angle to stretch the DNA fibers onto the slide. The resulting DNA spreads were air-dried, fixed in 3:1 methanol/acetic acid, and stored at 4 °C overnight. The DNA fibers were denatured by incubating them in 2.5 M HCl for 1 h at RT, washed five times with PBS and blocked with 2% BSA in PBST (PBS and Tween 20) for 40 min at RT. The newly replicated CldU and IdU tracks were stained for 2.5 h at RT using two different anti-BrdU antibodies recognizing CldU (Abcam, ab6326, 1:500) and IdU (Becton Dickinson, 347580, 1:100), respectively. After washing five times with PBST (PBS and Tween 20) the slides were stained with Anti-mouse Alexa 488 (Invitrogen, A-11001, 1:300) and anti-rat Cy3 (Immuno Research, 712-166-1530, 1:150) secondary antibodies for 1 h at RT in the dark. The slides were mounted in 30 μL Prolong Gold antifade reagent (Invitrogen). For S1 nuclease experiments, cells were treated as described above, labeled with CldU for 30 min, with IdU for 30 min, and incubated with S1 nuclease for 30 min as described^10^.

Microscopy was done using a Leica DM6 B microscope (HCX PL APO 63× objective). To assess fork progression the IdU/CldU ratio or IdU track lengths of at least 100 fibers per sample were measured using the line tool in ImageJ64 software. Statistical analysis was carried out using GraphPad Prism 7.

### Isolation of proteins on nascent DNA or iPOND

iPOND was performed as originally described^50^ with minor modifications. HEK293T cells were labeled with 10 µM EdU (Life Technologies) for 10 min and treated with the different drugs as indicated in Supplementary Fig. 2a. For the pulse-chase experiments with thymidine, after EdU incorporation, cells were washed with cell culture medium and incubated for 50 min in medium supplemented with 10 µM thymidine (Sigma-Aldrich). Cells were cross-linked with 1% formaldehyde for 20 min at RT, quenched with 0.125 M glycine for 5 min, and washed three times with cold PBS. For the conjugation of EdU with biotin azide, cells were permeabilized with 0.25% Triton X-100/PBS for 30 min, washed twice with PBS, and incubated in click reaction buffer (10 mM sodium-l-ascorbate, 20 µM biotin azide (Life Technologies), and 2 mM CuSO_4_ at RT for 2 h on a rotator). DMSO was used instead of biotin azide for the “no click control”. Cells were washed twice with PBS, resuspended in lysis buffer (50 mM Tris-HCl, pH 8.0, and 1% SDS) supplemented with protease inhibitors (Sigma-Aldrich), and chromatin was solubilized by sonication in a Bioruptor (Diagenode) at 4 °C at the highest setting for 10 min (30 s on and 30 s off cycles). After centrifugation for 10 min at 16,000 RCF, supernatants were diluted with 1:1 PBS (vol/vol) containing protease inhibitors and incubated overnight with streptavidin-agarose beads (EMD Millipore). Beads were washed once with lysis buffer, once with 1 M NaCl, twice with lysis buffer, and once with PBS, and captured proteins were eluted by boiling beads in 2× NuPAGE LDS Sample Buffer (Life Technologies) containing 100 mM DTT for 45 min at 95 °C. Proteins were resolved by electrophoresis using NuPAGE Novex 4–20% Bis-Tris gels and detected by Western blotting with the indicated antibodies: rabbit anti-Histone H3 (1:10000, abcam; ab1791), mouse anti-Pcna (1:2000, Santa-Cruz; sc-56), mouse anti-Actin (1:1000, Abcam; A5441), rabbit anti-Smarcal1 (1:1000, abcam; ab2559972), rabbit anti-Rad51 (1:1000, BioAcademia; 70-001).

### Metaphase spread analysis

U2OS cells were treated with 100 nM CPT for 2 h. Where indicated, cells were pretreated with 100 nM LatB 10 min prior to CPT and maintained during the genotoxic treatment. RPE-1 cells stably expressing NLS-Actin-WT or NLS-Actin-R62D were induced with 1 μM/mL Dox 24 h before performing the experiment. Cells were then treated with 50 nM CPT for 8 h. Genotoxic agents were removed by washing three times with 1 × PBS and the cells were then released into fresh medium containing 200 ng ml^−1^ nocodazole for 16 h. Cells were collected and swollen with 75 mM KCl for 20 min at 37 °C. Swollen mitotic cells were collected and fixed with methanol:acetic acid (3:1). The fixing step was repeated two times. Cells were then dropped onto pre-hydrated glass slides and air-dried overnight. The following day, slides were mounted with Vectashield medium containing DAPI. Images were acquired with a microscope (model DMRB; Leica) at 63x magnification equipped with a camera (model DFC360 FX; Leica) and visible chromosome abnormalities per metaphase spread were counted.

### Electron microscopic analysis of genomic DNA

Asynchronous and subconfluent U2OS cells were treated with 100 nM CPT for 1 h. Where indicated, cells were pretreated with 100 nM LatB or Swi 10 min prior to CPT and maintained during the genotoxic treatment. Asynchronous and subconfluent RPE-1 cells stably expressing NLS-Actin-WT or NLS-Actihn-R62D were induced with 1 μM/mL Dox 24 h before collecting the cells. Cells were then treated with 100 nM CPT or 20 nM ETP for 1 h. Cells were collected, resuspended in ice-cold PBS and crosslinked with 4,5′, 8-trimethylpsoralen (10 μg/ml final concentration), followed by irradiation pulses with UV 365 nm monochromatic light (UV Stratalinker 1800; Agilent Technologies). For DNA extraction, cells were lysed (1.28 M sucrose, 40 mM Tris-HCl [pH 7.5], 20 mM MgCl2, and 4% Triton X-100; Qiagen) and digested (800 mM guanidine–HCl, 30 mM Tris-HCl [pH 8.0], 30 mM EDTA [pH 8.0], 5% Tween-20, and 0.5% Triton X-100) at 50 °C for 2 h in presence of 1 mg/ml proteinase K. The DNA was purified using chloroform/isoamylalcohol (24:1) and precipitated in one volume of isopropanol. Finally, the DNA was washed with 70% EtOH and resuspended in 200 μl TE (Tris-EDTA) buffer. 120 U of restriction enzyme (PvuII high fidelity, New England Biolabs) were used to digest 6 μg of the purified genomic DNA for 5 h at 37 C. RNase A (Sigma–Aldrich, R5503) to a final concentration of 250 μg/ml was added for the last 2 h of this incubation. The digested DNA purified using the Thermo Fisher Silica Bead Gel Extraction kit according to manufacturer’s instructions. The Benzyldimethylalkylammonium chloride (BAC) method was used to spread the DNA on the water surface and then load it on carbon-coated 400-mesh nickel grids (G2400N, Plano Gmbh). Subsequently, DNA was coated with platinum using a High Vacuum Evaporator BAF060 (Leica). The grids were imaged automatically using a Talos 120 transmission electron microscope (FEI; LaB6 filament, high tension ≤120 kV) with a bottom-mounted CMOS camera BM-Ceta (4000 × 4000 pixel) and the MAPS software package (Thermo Fisher Scientific, Eindhoven, The Netherlands) as described^55^. Samples were annotated for replication intermediates using the MAPS Viewer software, overlapping images for annotated regions were stitched together using the automated pipeline ForkStitcher^55^ and final images were analyzed using ImageJ. For each experimental condition at least 70 replication fork molecules were analyzed in two to three different biological replicates.

### Immunofluorescence and imaging of fixed samples

U2OS cells transiently transfected with FLAG-NLS-WT or FLAG-NLS-R62D-Actin for 24 h or stably expressing FLAG-NLS-Actin were grown on sterile 12-mm diameter glass coverslip coated with poly-l-lysine, incubated for 4 min with 10 μM EdU, washed with 1× PBS and pre-extracted for 10 min with CSK-buffer (10 mM PIPES, 50 mM NaCl, 300 mM Sucrose, 3 mM MgCl_2_, 1 mM EGTA, and 0.5% Triton X-100) on ice, fixed in 4% buffered paraformaldehyde, washed three times with 1× PBS, permeabilized for 10 min at room temperature in 0.3% Triton X-100 (Sigma-Aldrich) in PBS and washed twice in PBS. EdU detection was performed with a Click-iT Plus EdU Alexa-Fluor 555 Imaging Kit according to the manufacturer’s recommendations (Thermo Fisher Scientific) before incubation with primary antibodies. All primary and secondary antibodies were diluted in 3% BSA/PBS. Incubation with primary antibodies was performed at room temperature for 2 h or overnight at 4 °C. Coverslips were washed three times with PBS containing 0.1% Tween-20 (Sigma-Aldrich). Secondary-antibody incubations were performed at room temperature for 45 min. After one wash with PBS containing 0.1% Tween-20 (Sigma-Aldrich) and one with PBS, coverslips were incubated for 10 min with PBS containing DAPI (0.5 mg/mL) at room temperature to stain DNA. Following three washing steps in PBS, coverslips were briefly washed with distilled water, dried on 3 mm paper and mounted with Prolong Gold antifade reagent (Invitrogen). Imaging with the Deltavision microscope and image processing (deconvolution) for fixed cells done in Figs. 1, 2a, b, Supplementary Fig. 1o, p was done as in ref. ^40^. Confocal microscopy of a single middle *Z-*stack from fixed S phase (EdU+) U2OS cells in Supplementary Fig. 1k, l was done using a TCS SP5 Leica confocal microscope equipped with a pulsed white light laser and an HCX PL APO ×63 oil-immersion objective (NA, 1.4).

### Live cell imaging

Cells expressing PCNA-CB-RFP (Chromotek, ccr) and nAC-GFP were seeded on a previously poly-L-lysine coated μ-slides glass-bottom dish (Ibidi). Culture medium was replaced with colourless DMEM (FluoroBrite DMEM, ThermoFisher Scientific) 2 h before imaging. Imaging was performed using the incubation system for live cell imaging (cellVIVO) at 37 °C and 5% CO_2_ on an Olympus IXplore SpinSR10 (Olympus Europe SE & Co. KG, Germany) with a Yokogawa CSU-SoRa disk and UPLSAPO 100×/1.3 NA oil-immersion objective. In order to avoid phototoxicity and photobleaching, images were acquired every 20 s (imaging frames) for three time intervals of 5 min (T1, T2, T3 in the figures) separated by 5 min dark intervals. Z series were collected with 0.3 μm intervals over a 12 μm range. 200 nM ETP or H2O (NT) were added to the cells just before imaging. Mitotic cells were also imaged every 20 s until detectable exit from mitosis, whereas those treated with 750 nM A23817 were imaged every minute until clear F-actin dissolution.

For experiments with the LifeAct or F-tractin probe, U2OS cells were seeded onto μ-slide 8 well glass-bottom chambers and transiently transfected with LifeAct-GFP-NLS or F-tractin-GFP-NLS using Lipofectamine 3000 according to manufacturer´s instructions 24 h before imaging. 20 nM ETP was added to the cells just before imaging using a LSM800 confocal laser-scanning microscope (Zeiss) equipped with a 63×, 1.4 NA oil objective. Images were acquired every 50 s and processed with the Zen blue software (Zeiss).

### MSD analysis

PCNA foci were tracked in 3D using a semi-automated method and manually corrected to ensure optimal connections between time points with a technique similar to as previously described for DNA damage foci^75,76^. Tracked foci were used to register the cells. After registration, PCNA foci were retracked and focus positional data were extracted in Excel and analyzed in Matlab (MathWorks) to calculate MSDs as previously described^75,76^.

### Image processing

Images for Supplementary Fig. 1c–e were collected with a spinning disc confocal, visualized as volumes in Imaris before image preparation. Images for Supplementary Fig. 1k–l were collected with a confocal microscope as a single Z-stack to visualize intranuclear structures specifically. Images for Figs. 1 and 2 were collected with a Deltavision microscope, deconvolved 5 times with a conservative algorithm, and displayed as maximum intensity projections. Videos were generated using individual snapshots from volume reconstruction, saved as.tiff files and assembled in Fiji.

### Image analyses

In Fig. 1a, b, cells were scored as positive when nuclear actin structures were detected and as negative when no nuclear actin structures were observed. Cells with nuclear actin structures were further classified into subcategories as follows: ‘Foci only’ are cells solely displaying punctate structures of actin as described in Fig. 1a and Supplementary Fig. 1o. ‘Foci+patches/filaments’ contain a combination of foci with other F-actin structures for each cell. Cells were classified as G1 or S based on nuclear size (determined by DAPI) and the presence of EdU staining. For the calculation of actin filaments in Supplementary Fig. 1g, only new filaments that were not present in the previous frame were counted, for each time point. Quantification of the colocalization of Edu and actin foci in Fig. 2a was performed in Imaris 9.5.0 using the ‘Spots Detection’ function. Spots corresponding to EdU and to actin were independently generated using a threshold value of 0.3 µm. Colocalizing signals were defined using the ‘Spot Colocalize’ extension and identified based on a maximum distance between the centers of 0.4 µm spots. Figures were generated in Imaris and Photoshop. Measurements of the actin filaments length used in Fig. 1a, c and Supplementary Fig. 1o, p was performed in Imaris 9.5.0 using the ‘MeasurementPro’ module to calculate the distance between the two ends of each filament in 3D images.

### Reporting summary

Further information on research design is available in the Nature Portfolio Reporting Summary linked to this article.

### Supplementary information

Supplementary Information

Peer Review File

Description of Additional Supplementary Files

Supplementary Data 1

Supplementary Data 2

Supplementary Movie 1

Supplementary Movie 2

Supplementary Movie 3

Reporting Summary

### Source data

Source Data

## Data Availability

Raw data used to build all graphs and derive statistics - as well as original, uncropped blots - are available in the provided Source data file. Microscopy images are in the range of several Terabytes and would anyway require a trained eye for interpretation. They will hence be made available upon reasonable request.

## References

[1] Saxena S, Zou L (2022). Hallmarks of DNA replication stress. Mol. Cell.

[2] Macheret M, Halazonetis TD (2015). DNA replication stress as a hallmark of cancer. Annu. Rev. Pathol..

[3] Forment JV, O’Connor MJ (2018). Targeting the replication stress response in cancer. Pharmacol. Ther..

[4] Baillie KE, Stirling PC (2021). Beyond kinases: targeting replication stress proteins in cancer Therapy. Trends Cancer.

[5] Berti M, Cortez D, Lopes M (2020). The plasticity of DNA replication forks in response to clinically relevant genotoxic stress. Nat. Rev. Mol. Cell Biol..

[6] Neelsen KJ, Lopes M (2015). Replication fork reversal in eukaryotes: from dead end to dynamic response. Nat. Rev. Mol. Cell Biol..

[7] Quinet A, Lemacon D, Vindigni A (2017). Replication fork reversal: players and guardians. Mol. Cell.

[8] Betous R (2012). SMARCAL1 catalyzes fork regression and Holliday junction migration to maintain genome stability during DNA replication. Genes Dev..

[9] Vujanovic M (2017). Replication fork slowing and reversal upon DNA damage require PCNA polyubiquitination and ZRANB3 DNA translocase activity. Mol. Cell.

[10] Bai, G. et al. HLTF promotes fork reversal, limiting replication stress resistance and preventing multiple mechanisms of unrestrained DNA synthesis. *Mol. Cell* (2020) 10.1016/j.molcel.2020.04.031. 10.1016/j.molcel.2020.04.031 PMC7305998 32442397

[11] Zellweger R (2015). Rad51-mediated replication fork reversal is a global response to genotoxic treatments in human cells. J. Cell Biol..

[12] Liu W (2023). RAD51 bypasses the CMG helicase to promote replication fork reversal. Science.

[13] Mijic S (2017). Replication fork reversal triggers fork degradation in BRCA2-defective cells. Nat. Commun..

[14] Lemacon D (2017). MRE11 and EXO1 nucleases degrade reversed forks and elicit MUS81-dependent fork rescue in BRCA2-deficient cells. Nat. Commun..

[15] Chaudhuri AR (2016). Replication fork stability confers chemoresistance in BRCA-deficient cells. Nature.

[16] Guilliam TA, Doherty AJ (2017). PrimPol-prime time to reprime. Genes.

[17] Mourón S (2013). Repriming of DNA synthesis at stalled replication forks by human PrimPol. Nat. Struct. Mol. Biol..

[18] Bianchi J (2013). PrimPol bypasses UV photoproducts during eukaryotic chromosomal DNA replication. Mol. Cell.

[19] Piberger AL (2020). PrimPol-dependent single-stranded gap formation mediates homologous recombination at bulky DNA adducts. Nat. Commun..

[20] González‐Acosta D (2021). PrimPol‐mediated repriming facilitates replication traverse of DNA interstrand crosslinks. EMBO J..

[21] Tirman S (2021). Temporally distinct post-replicative repair mechanisms fill PRIMPOL-dependent ssDNA gaps in human cells. Mol. Cell.

[22] Quinet A (2020). PRIMPOL-mediated adaptive response suppresses replication fork reversal in BRCA-deficient cells. Mol. Cell.

[23] Jacobs K (2022). Stress-triggered hematopoietic stem cell proliferation relies on PrimPol-mediated repriming. Mol. Cell.

[24] Mutreja K (2018). ATR-mediated global fork slowing and reversal assist fork traverse and prevent chromosomal breakage at DNA interstrand cross-links. Cell Rep..

[25] Wootton J, Soutoglou E (2021). Chromatin and nuclear dynamics in the maintenance of replication fork integrity. Front. Genet..

[26] González-Acosta, D. & Lopes, M. DNA replication and replication stress response in the context of nuclear architecture. *Chromosoma*10.1007/s00412-023-00813-7 (2023). 10.1007/s00412-023-00813-7 PMC10904558 38055079

[27] Klages-Mundt NL, Kumar A, Zhang Y, Kapoor P, Shen X (2018). The nature of actin-family proteins in chromatin-modifying complexes. Front. Genet..

[28] Ulferts S, Prajapati B, Grosse R, Vartiainen MK (2021). Emerging properties and functions of actin and actin filaments inside the nucleus. CSH Perspect. Biol..

[29] Hurst V, Shimada K, Gasser SM (2019). Nuclear actin and actin-binding proteins in DNA repair. Trends Cell Biol..

[30] de Lanerolle P, Serebryannyy L (2011). Nuclear actin and myosins: life without filaments. Nat. Cell Biol..

[31] Melak M, Plessner M, Grosse R (2017). Actin visualization at a glance. J. Cell Sci..

[32] Caridi CP, Plessner M, Grosse R, Chiolo I (2019). Nuclear actin filaments in DNA repair dynamics. Nat. Cell Biol..

[33] Lamm N, Rogers S, Cesare AJ (2021). Chromatin mobility and relocation in DNA repair. Trends Cell Biol..

[34] Baarlink C, Wang H, Grosse R (2013). Nuclear actin network assembly by formins regulates the SRF coactivator MAL. Science.

[35] Plessner M, Melak M, Chinchilla P, Baarlink C, Grosse R (2015). Nuclear F-actin formation and reorganization upon cell spreading. J. Biol. Chem..

[36] Wang Y (2019). GPCR-induced calcium transients trigger nuclear actin assembly for chromatin dynamics. Nat. Commun..

[37] Tsopoulidis, N. et al. T cell receptor–triggered nuclear actin network formation drives CD4+ T cell effector functions. *Sci. Immunol.* **4**, eaav1987 (2019). 10.1126/sciimmunol.aav1987 30610013

[38] Baarlink C (2017). A transient pool of nuclear F-actin at mitotic exit controls chromatin organization. Nat. Cell Biol..

[39] Belin BJ, Lee T, Mullins RD (2015). DNA damage induces nuclear actin filament assembly by Formin-2 and Spire-1/2 that promotes efficient DNA repair. Elife.

[40] Caridi CP (2018). Nuclear F-actin and myosins drive relocalization of heterochromatic breaks. Nature.

[41] Aymard F (2017). Genome wide mapping of long range contacts unveils DNA Double Strand Breaks clustering at damaged active genes. Nat. Struct. Mol. Biol..

[42] Schrank BR (2018). Nuclear Arp2/3 drives DNA break clustering for homology-directed repair. Nature.

[43] Parisis N (2017). Initiation of DNA replication requires actin dynamics and formin activity. EMBO J..

[44] Lamm N (2020). Nuclear F-actin counteracts nuclear deformation and promotes fork repair during replication stress. Nat. Cell Biol..

[45] Han S-S (2022). WASp modulates RPA function on single-stranded DNA in response to replication stress and DNA damage. Nat. Commun..

[46] Nieminuszczy J (2023). Actin nucleators safeguard replication forks by limiting nascent strand degradation. Nucleic Acids Res..

[47] Shi J (2023). Nuclear myosin VI maintains replication fork stability. Nat. Commun..

[48] Posern G, Sotiropoulos A, Treisman R (2002). Mutant actins demonstrate a role for unpolymerized actin in control of transcription by serum response factor. Mol. Biol. Cell.

[49] Kokai E (2014). Analysis of nuclear actin by overexpression of wild-type and actin mutant proteins. Histochem. Cell Biol..

[50] Sirbu BM (2011). Analysis of protein dynamics at active, stalled, and collapsed replication forks. Genes Dev..

[51] Hetrick, B., Han, M. S., Helgeson, L. A. & Nolen, B. J. Small Molecules CK-666 and CK-869 Inhibit Actin-Related Protein 2/3 Complex by Blocking an Activating Conformational Change. *Chem. Biol.* **20**, 701–712 (2013). 10.1016/j.chembiol.2013.03.019 PMC3684959 23623350

[52] Jackson DA, Pombo A (1998). Replicon clusters are stable units of chromosome structure: evidence that nuclear organization contributes to the efficient activation and propagation of S phase in human cells. J. Cell Biol..

[53] Zellweger, R. & Lopes, M. *Genome Instability* (Springer New York, 2017).

[54] Halder, S., Ranjha, L. Taglialatela, A., Ciccia, A. & Cejka, P. Strand annealing and motor driven activities of SMARCAL1 and ZRANB3 are stimulated by RAD51 and the paralog complex. *Nucleic Acids Res.*10.1093/nar/gkac583 (2022). 10.1093/nar/gkac583 PMC9371921 35801922

[55] Stoy H (2022). R-Loops, methods and protocols. Methods Mol. Biol..

[56] Wang Y-H (2017). DNA damage causes rapid accumulation of phosphoinositides for ATR signaling. Nat. Commun..

[57] Berti M (2013). Human RECQ1 promotes restart of replication forks reversed by DNA topoisomerase I inhibition. Nat. Struct. Mol. Biol..

[58] Maya-Mendoza A (2018). High speed of fork progression induces DNA replication stress and genomic instability. Nature.

[59] Kang Z (2021). BRCA2 associates with MCM10 to suppress PRIMPOL-mediated repriming and single-stranded gap formation after DNA damage. Nat. Commun..

[60] Quinet A, Carvajal-Maldonado D, Lemacon D, Vindigni A (2017). DNA fiber analysis: mind the gap!. Methods Enzymol..

[61] Mehta KPM (2022). CHK1 phosphorylates PRIMPOL to promote replication stress tolerance. Sci. Adv..

[62] Mansilla SF (2023). Polymerase iota (Pol ι) prevents PrimPol-mediated nascent DNA synthesis and chromosome instability. Sci. Adv..

[63] Knerr, J. et al. Formin-mediated nuclear actin at androgen receptors promotes transcription. *Nature* 1–3. 10.1038/s41586-023-05981-1 (2023). 10.1038/s41586-023-05981-1 36972684

[64] Guilliam TA (2017). Molecular basis for PrimPol recruitment to replication forks by RPA. Nat. Commun..

[65] Berti M (2020). Sequential role of RAD51 paralog complexes in replication fork remodeling and restart. Nat. Commun..

[66] Gaggioli V (2023). Dynamic de novo heterochromatin assembly and disassembly at replication forks ensures fork stability. Nat. Cell Biol..

[67] Wynne DJ, Rog O, Carlton PM, Dernburg AF (2012). Dynein-dependent processive chromosome motions promote homologous pairing in *C. elegans* meiosis. J. Cell Biol..

[68] Nagai S (2008). Functional targeting of DNA damage to a nuclear pore-associated SUMO-dependent ubiquitin ligase. Science.

[69] Su XA, Dion V, Gasser SM, Freudenreich CH (2015). Regulation of recombination at yeast nuclear pores controls repair and triplet repeat stability. Gene Dev..

[70] Cho NW, Dilley RL, Lampson MA, Greenberg RA (2014). Interchromosomal homology searches drive directional ALT telomere movement and synapsis. Cell.

[71] Dion V, Kalck V, Horigome C, Towbin BD, Gasser SM (2012). Increased mobility of double-strand breaks requires Mec1, Rad9 and the homologous recombination machinery. Nat. Cell Biol..

[72] Miné-Hattab J, Rothstein R (2012). Increased chromosome mobility facilitates homology search during recombination. Nat. Cell Biol..

[73] Oshidari R (2018). Nuclear microtubule filaments mediate non-linear directional motion of chromatin and promote DNA repair. Nat. Commun..

[74] Kyheröinen S, Vartiainen MK (2020). Nuclear actin dynamics in gene expression and genome organization. Semin. Cell Dev. Biol..

[75] See C, Arya D, Lin E, Chiolo I (2020). Live cell imaging of nuclear actin filaments and heterochromatic repair foci in drosophila and mouse cells. Methods Mol. Biol. Clifton N J..

[76] Caridi CP (2018). Quantitative methods to investigate the 4D dynamics of heterochromatic repair sites in Drosophila cells. Methods Enzymol..

