# Supplementary material for: Nuclear actin polymerization rapidly mediates replication fork remodeling upon stress by limiting PrimPol activity
Source: Nat Commun. 2023 Nov 28;14:7819. doi: 10.1038/s41467-023-43183-5 (PMC10684888; doi:10.1038/s41467-023-43183-5)
Supplement: Supplementary file 1 — Supplementary Information [file 41467_2023_43183_MOESM1_ESM.pdf]

Supplementary Figures and legends

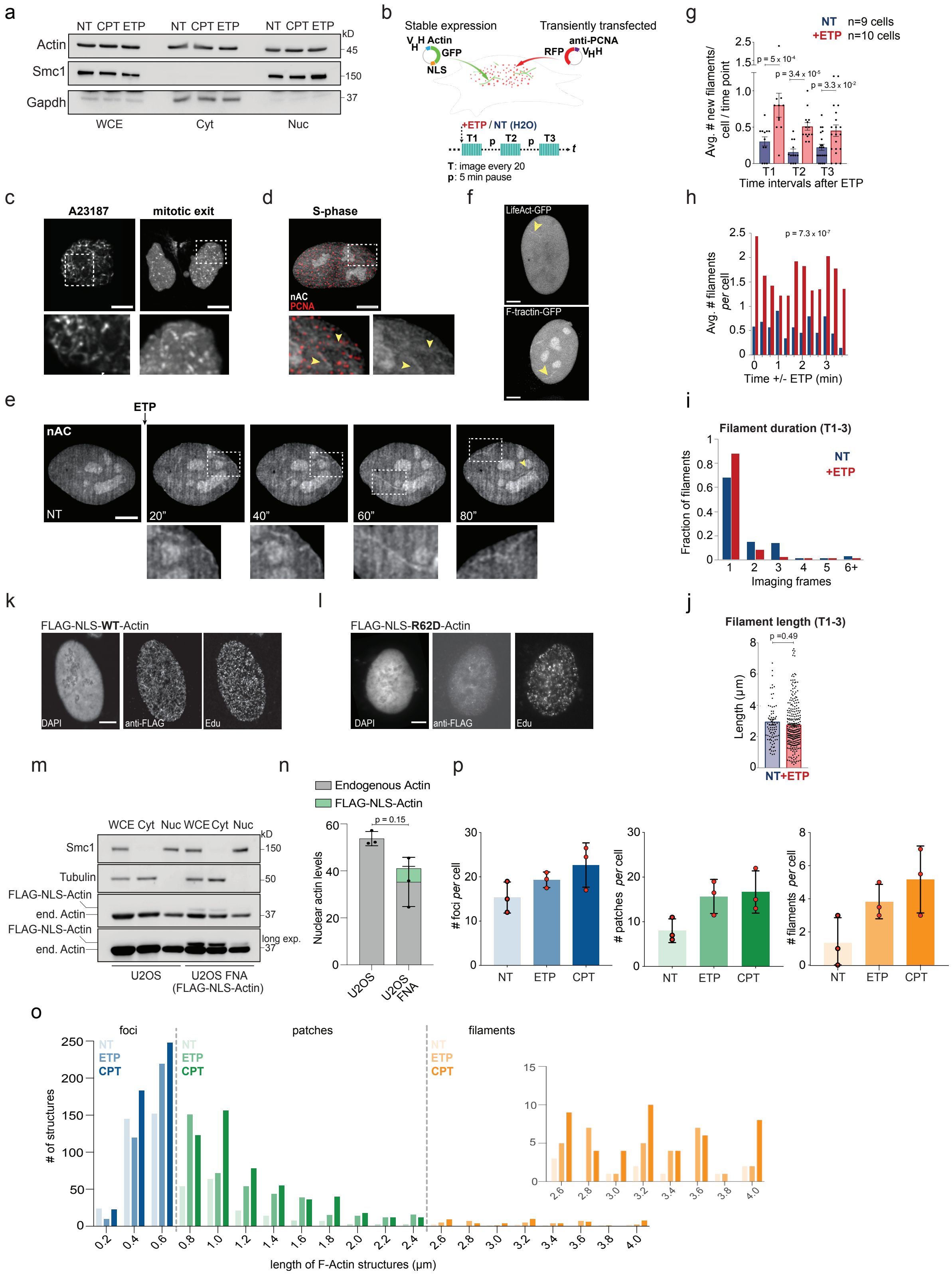

Supplementary Figure 1. Nulcear F-actin visualization via live-cell imaging or fixed-cell staining (related to Fig. 1). **a**. Western blot depicting whole cell extracts (WCE), cytosolic (Cyt) or nuclear (Nuc) actin protein levels in cells stably expressing FLAG-NLS-Actin optionally treated with 100 nM CPT or 20 nM ETP for 1 hour. Smc1 and Gapdh were used as fractionation and loading controls. The experiment was repeated three times yielding similar results (data not shown). **b**. Top: experimental setup for live-cell imaging: U2OS cells stably expressing nAC-GFP (nuclear actin-chromobody-GFP) were transiently transfected with PCNA-CB-RFP (PCNA-chromobody-RFP) to monitor actin dynamics in replicating cells. Bottom: experimental scheme of the time-lapse imaging experiment in untreated cells (NT: H2O) or cells treated with 200nM Etoposide (+ETP). Images were taken every 20 seconds for three time intervals (T1, T2, T3) separated by 5 min dark intervals with a spinning-disk confocal microscope. **c**. Representative images from spinning-disk confocal live microscopy analysis of U2OS cells stably expressing nAC-GFP. Cells were imaged immediately after treatment with calcium ionophore A23187 (750 nM) or during mitotic exit, as indicated. See also Supplementary Movies 1-2. **d**. Representative image from spinning-disk confocal live microscopy analysis of S-phase U20S cell (transiently transfected with PCNA-CB-RFP) stably expressing nAC-GFP. Zoomed details highlight examples of transient nuclear actin filaments in PCNA+ cells (yellow arrow in the zoomed detail). **e.** Representative images from spinning-disk confocal live microscopy analysis of PCNA+ U20S cell stably expressing nAC-GFP at indicated time points before (NT) or after 200 nM ETP treatment. Zoomed details highlight examples of transient nuclear actin filaments. See also Supplementary Movie 3. **f.** Representative images from confocal laser scanning live microscopy analysis of PCNA+ U20S cell transiently expressing LifeAct-GFP (top) or F-tractin-GFP (bottom) after 20 nM ETP treatment. **e, f.** Yellow arrows indicate transient filaments. **g**. Quantification shows the average number of new actin filaments forming in NT or ETP-treated cells for each cell and time point, during each of the three time intervals described in b. Bar graphs are mean +/-S.E.M. n=9 cells for the NT and n=10 cells for +ETP from 3 or more independent experiments. **h.** Average number of filaments *per* cell during the first time point (T1 – immediately after optional ETP addition) as described in b. Data are mean ± S.E.M, N=84 filaments (9 cells) for UNT and N= 273 filaments (10 cells) for +ETP **i.** Duration of filaments in untreated cells (NT) or cells treated with 200nM Etoposide (+ETP) from the experiment described in Extended Figure 1b. Time points were collected every 20 sec. **j.** Actin filament length observed in NT or ETP treated cells from the experiment described in b. Data are mean ± S.E.M, N=84 filaments (9 cells) for NT and N= 273 filaments (10 cells) for +ETP in **h, I** and **j.** Statistical analysis: numerical P-values for the indicated comparisons were calculated with two-tailed Mann–Whitney test in g, h and j. **k.** Confocal microscopy of a single middle *Z*-stack from fixed S-phase (EdU+) U2OS cell transiently transfected with FLAG-NLS-WT-Actin and stained for FLAG and EdU. **l.** Representative confocal microscopy images of fixed S-phase (EdU+) U2OS cells transiently transfected with FLAG-NLS-R62D-Actin and stained for FLAG and EdU. **c-f,k and l.** Scale bars=5 µm. **m.** Western blot showing whole cell extracts (WCE), cytosolic (Cyt) or nuclear (Nuc) actin levels in U2OS WT cells stably expressing FLAG-NLS-Actin. Smc1 and Tubulin were used as fractionation and loading controls. Actin antibody serves to distinguish endogenous vs exogenous actin**. n.** quantification of m. Bar graph depicts mean and SD of quantified nuclear Actin protein levels from three independent experiments. Values were normalized to the WCE signal, previously corrected by the Smc1 signal. Statistical analysis: two-way ANOVA, applying Geisser-Greenhouse correction and compared with Bonferroni's test. **o**. Bin distribution of the length of F-Actin structures (in µm) used to set defined thresholds for the three size categories analyzed in Figure 1a – c. **p.** Medians of three biological replicates of the analysis of the number of actin structures displayed in Figure 1c are represented as red dots. Bar represents the mean +/- SD. Source data are provided as a Source Data file.

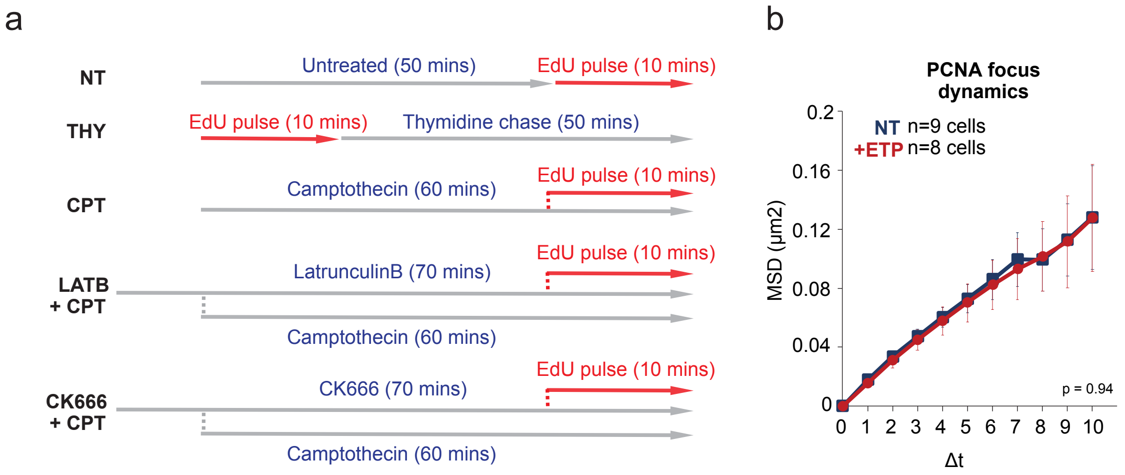

Supplementary Figure 2. iPOND lebelling scheme and mobility of PCNA foci (related to Fig. 2). **a**. Labeling scheme for the iPOND experiment in Fig 2b and 4a. HEK293T cells were labeled with 10 µM EdU for 10 minutes (NT) followed by a chase into 10 µM thymidine (Thy) for 50 minutes. In the CPT sample, cells were treated with 100 nM CPT for 60 minutes and EdU was added in the last 10 minutes. Cells were optionally treated with 100 nM LatB 10 minutes prior to CPT treatment (LatB+CPT) and maintained during the genotoxic treatment. **b**. MSD analysis of PCNA foci in cells transiently transfected with PCNA-CB-RFP after indicated treatments (N=2376 foci and n=9 cells for NT; N=2105 foci and n=8 cells for +ETP from 3 or more independent experiments). Data are mean ± SEM. Statistical analysis: p-values were calculated with extra sum-of-squares *F*-test, nonlinear regression for curve fitting. Δ*t*, time intervals (intervals were 20 s each). Source data are provided as a Source Data file.

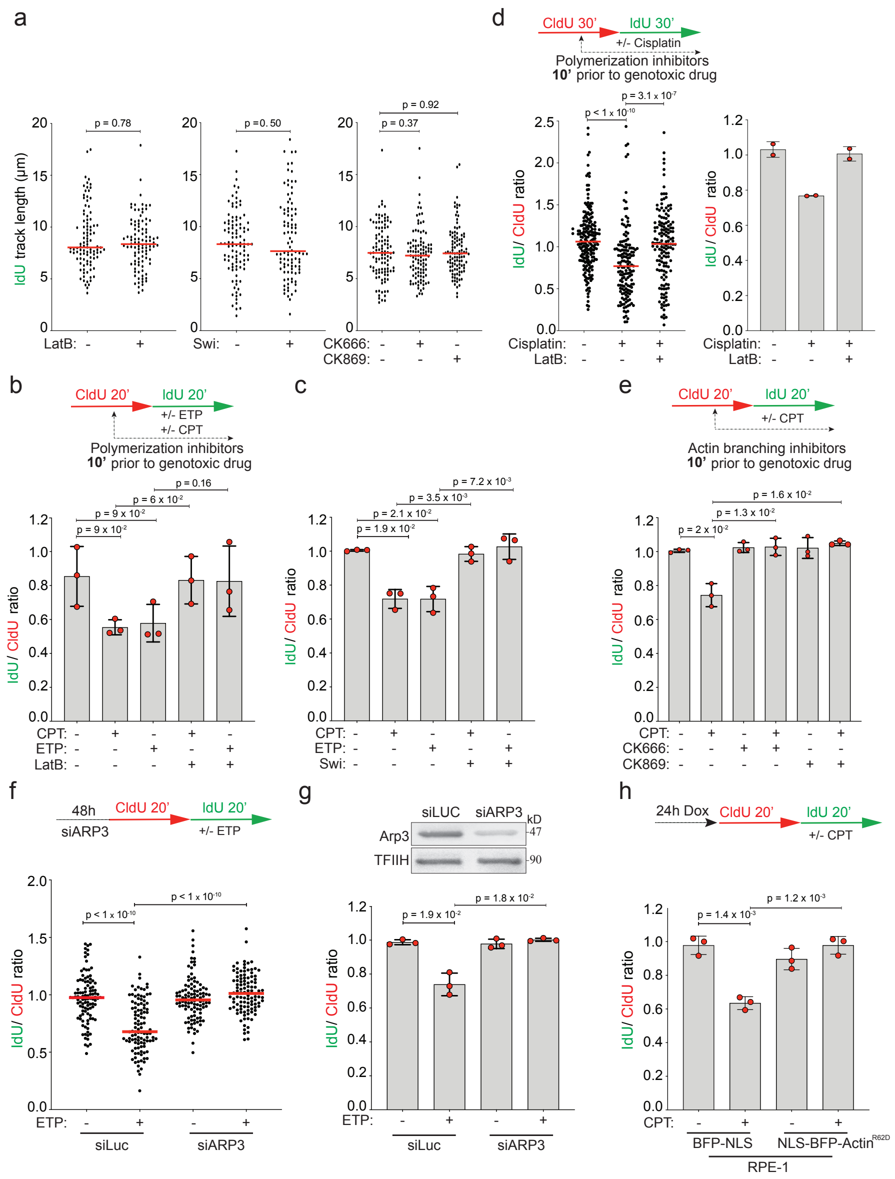

Supplementary Figure 3. Impact of inactivating nuclear actin polymerization on DNA damage-induced fork slowing (related to Fig. 3). **a**. DNA fiber analysis of U2OS cells. IdU track length (µm) is plotted for a minimum of 100 forks (indicated as black dots) per sample from a single, representative experiment. Red lines indicate the median. Statistical analysis: Mann–Whitney test. Three independent experiments yielded similar results (data not shown). **b-c, e**. Medians of three biological replicates for experiments displayed in Fig. 3b-d are represented as red dots. Bar graphs represent mean +/- SD. 100nM LatB, Swi, CK666 or CK869 were added 10 minutes prior to 100 nM CPT or 20 nM ETP and were retained during the IdU labelling. **d.** Top: CldU/IdU pulse-labeling protocol used to evaluate fork progression upon 20 µM Cisplatin. Bottom left: IdU/CIdU ratio is plotted as a readout of fork progression. Statistical analysis: Mann–Whitney test. Bottom right: medians of three biological fibers replicates are represented as red dots. Bar graph depicts mean +/- SD. **f**. Top: CldU/IdU pulse-labeling protocol used to evaluate fork progression upon 20 nM ETP. U2OS cells were transfected with siARP3 48 hours before CldU and IdU labeling. Bottom: IdU/CIdU ratio is plotted as a readout of fork progression. Statistical analysis: Mann–Whitney test. **g.** Top: Arp3 protein levels assessed by western blot. TFIIH, loading control. The experiment was performed three times yielding similar results (data not shown). Bottom: Medians of three biological fibers replicates are represented as red dots. Bar graph represents mean +/- SD. **h.** Top: schematic of the CldU/IdU pulse-labeling protocol used to evaluate fork progression upon 100 nM CPT. Doxycycline (Dox) was added 24 hours before the CldU/IdU pulse-labeling protocol. Bottom: medians of three biological replicates of fibers from Fig.3f are represented as red dots. Bar graph represents the mean. **b-d, e, g-h**. A minimum of 100 forks was scored for each replicate. Statistical analysis: two tailed Welch's test. Source data are provided as a Source Data file.

a

U2OS	NT	CPT	LatB	LatB + CPT	Swi	Swi + CPT
% RF Exp #1	**9 (115)**	**18 (119)**	**1 (71)**	**8 (131)**	**4 (72)**	**2 (73)**
% RF Exp #2	**3 (129)**	**16 (85)**	**1 (98)**	**4 (92)**	**3 (81)**	**5 (78)**
% RF Exp #3	**6 (71)**	**16 (87)**	**5 (76)**	**1 (74)**	**5 (73)**	**3 (78)**

b

RPE-1	BFP-NLS			NLS-BFP-Actin^{R62D}		
	NT	CPT	ETP	NT	CPT	ETP
% RF Exp #1	**8 (72)**	**22 (72)**	**18 (71)**	**4 (71)**	**7 (91)**	**3 (75)**
% RF Exp #2	**7 (91)**	**26 (72)**	**16 (75)**	**2 (88)**	**3 (79)**	**3 (105)**
% RF Exp #3	**5 (97)**	**16 (120)**	**11 (94)**	**5 (92)**	**5 (91)**	**3 (71)**

c

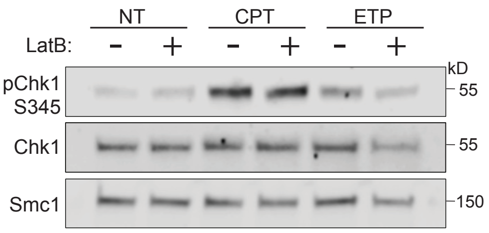

d

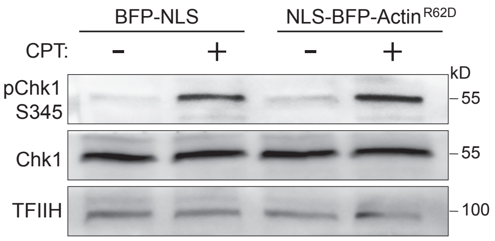

Supplementary Figure 4. Impact of defective nuclear F-actin on ATR signaling and on fork remodelling detected by EM (related to Fig. 4). **a-b.** Electron microscopy data summary tables. Frequency of observed reversed forks (% RF) in three independent experiments related to Figure 4e, f, respectively. Number of analyzed molecules is indicated in brackets. **c.** Western blot analysis of whole-cell extracts from U2OS cells, optionally treated for 1 hour with 20 nM ETP or 100 nM CPT. Where indicated, cells have been priorly treated with 100 nM LatB for 10 mins. **d**. Western blot analysis on whole-cell extracts from RPE-1 cells stably expressing doxycycline inducible BFP-NLS or NLS-BFP-Actin^{R62D}. Doxycycline (Dox) was added 24 hours before lysing the cells. BFP-NLS or NLS-BFP-Actin^{R62D} expressing cells were optionally treated for 1 hour with 100 nM CPT. **c-d.** The level of phosphorylated Chk1 was assessed using an antibody directed against pChk1-S345. The total Chk1 protein, Smc1 and TFIIH served as loading controls. The experiment was performed three times yielding similar results (data not shown). Source data are provided as a Source Data file.

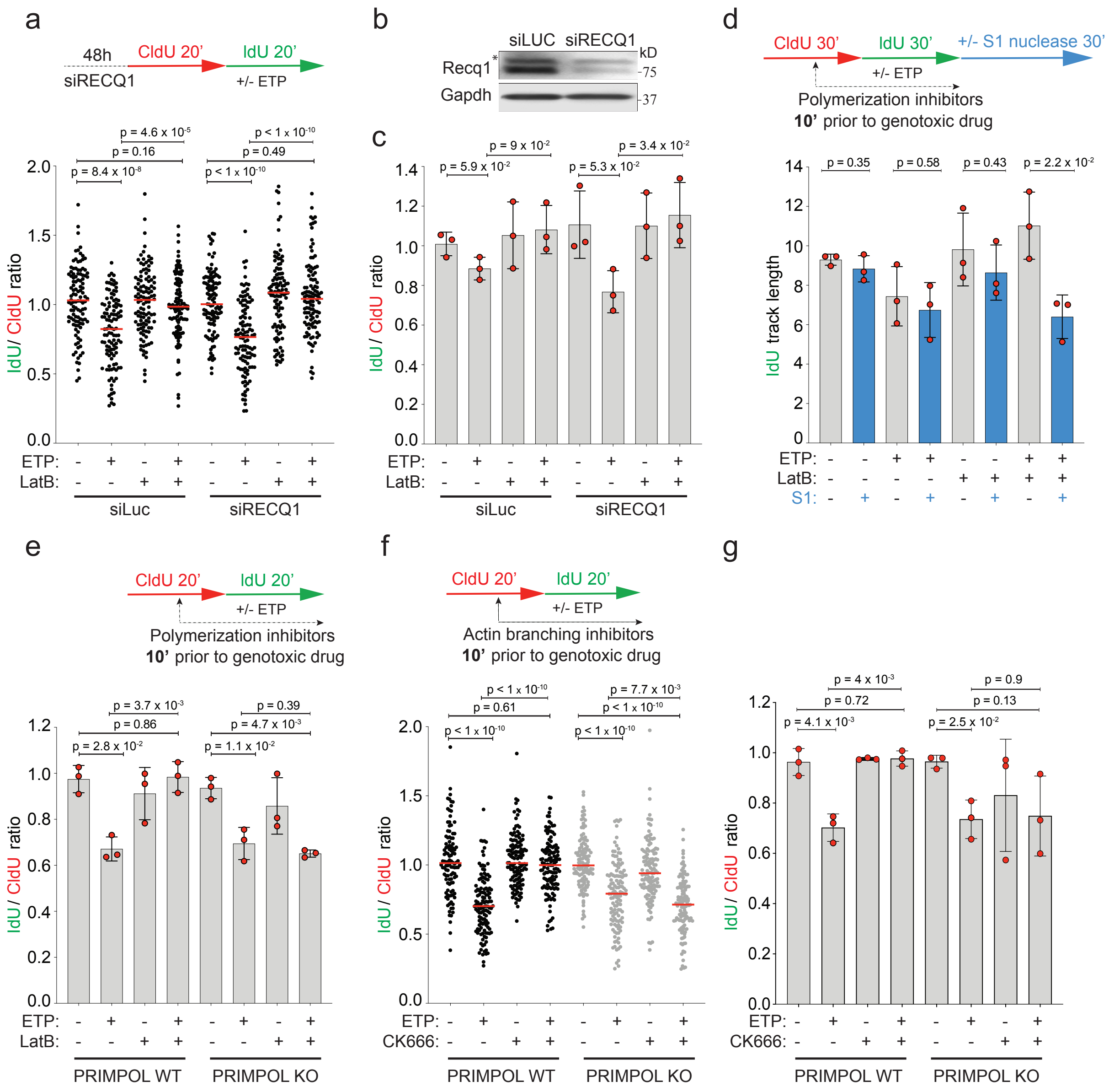

h

U2OS	PRIMPOL WT		PRIMPOL KO	
	ETP	LatB + ETP	ETP	LatB + ETP
% RF Exp #1	**18 (72)**	**7 (78)**	**14 (70)**	**13 (70)**
% RF Exp #2	**16 (71)**	**6 (76)**	**11 (70)**	**10 (70)**

Supplementary Figure 5. Role of RECQ1 and Primpol in fork progression and remodelling upon defective nuclear actin polymerization (related to Fig. 5). **a**. Top: CldU/IdU pulse-labeling protocol used to evaluate fork progression upon 20 nM ETP. U2OS cells were transfected with siRECQ1 48 hours before CldU and IdU labeling. Bottom: the IdU/CIdU ratio is plotted as a readout of fork progression for a minimum of 100 forks (indicated as black dots) per sample from a single, representative experiment. Red line indicates the median. Statistical analysis: Mann–Whitney test. **b**. RECQ1 levels assessed by western blot. GAPDH, loading control; *, unspecific band. The experiment was performed two times yielding similar results. **c.** Medians of three biological replicates from a are represented as red dots in Bar graph represents the mean. **c, d, e**, **g**. Medians of three biological replicates of fibers in Fig. 5b, e and Supplementary Fig. 5f are represented as red dots. The height of the bar represents the mean. A minimum of 100 forks was scored for each replicate. Statistical analysis: two tailed Welch's test. **f**. DNA fiber analysis of U2OS PRIMPOL WT and KO cells. Top: CldU/IdU pulse-labeling protocol used to evaluate fork progression upon 20 nM ETP. 100 nM CK666 was added 10 minutes prior to ETP and retained during the IdU labelling. Bottom: IdU/CIdU ratio is plotted as a readout of fork progression for a minimum of 100 per sample from a single, representative experiment. Statistical analysis: Mann–Whitney test. **h.** Electron microscopy data summary table. Frequency of observed reversed forks (% RF) in two independent experiments related to Figure 5f-g. Number of analyzed molecules is indicated in brackets. Source data are provided as a Source Data file.

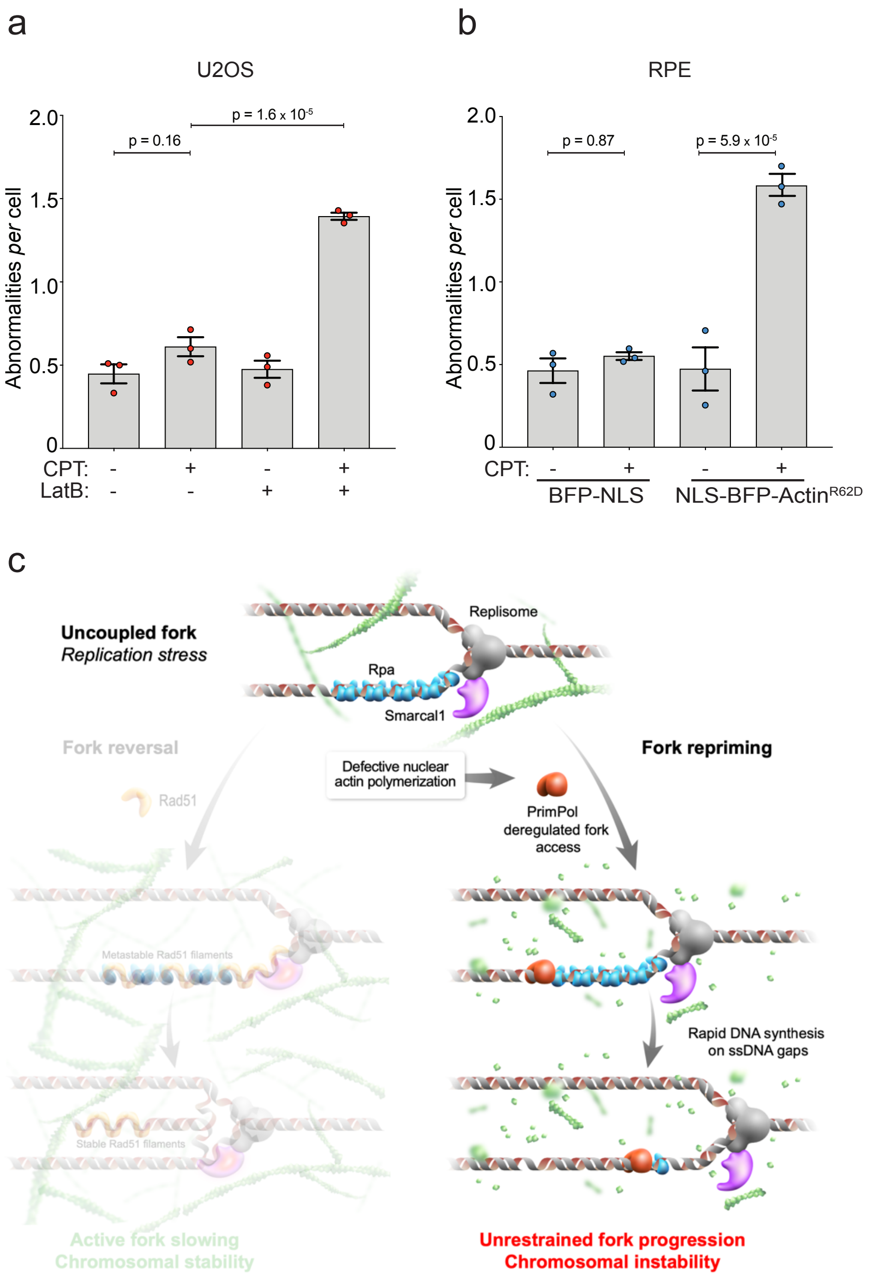

Supplementary Figure 6. Impact of defective nuclear actin polymerization on fork remodelling and chromosome stability (related to Fig. 6). **a.** Number of chromosomal abnormalities in U2OS cells optionally treated with 100 nM CPT for 2 hours. 100 nM LatB was added 10 minutes before CPT and retained during the genotoxic treatment. **b**. Number of chromosomal abnormalities in RPE-1 cells stably expressing doxycycline inducible NLS-Actin-WT or NLS-Actin-R62D. Doxycycline (Dox) was added for 24 hours prior to the experiment and cells were optionally treated for 8 hours with 50 nM CPT. **a**-**b**. Bar graph depicts mean +/- SD from three independent experiments (red and blue dots). A minimum of 50 metaphases was analyzed per sample. Statistical analysis: one-way ordinary ANOVA. **c.** Working hypothesis: Defective nuclear actin polymerization leads to deregulated PrimPol recruitment, promoting rapid DNA synthesis on ssDNA gaps at replication forks and accumulation of postreplicative ssDNA gaps. These conditions of unrestrained, discontinuous DNA synthesis ultimately lead to chromosomal instability (see also Fig. 6c and Discussion for details). Source data are provided as a Source Data file.
