# Supplementary material for: Nuclear actin polymerization rapidly mediates replication fork remodeling upon stress by limiting PrimPol activity
Source: Nat Commun. 2023 Nov 28;14:7819. doi: 10.1038/s41467-023-43183-5 (PMC10684888; doi:10.1038/s41467-023-43183-5)
Supplement: Supplementary file 3 — Description of Additional Supplementary Files [file 41467_2023_43183_MOESM3_ESM.pdf]

Description of Additional Supplementary Files

File Name: Supplementary Data 1
Description: List of antibodies used in this study.

File Name: Supplementary Data 2
Description: List of specific chemicals used in this study.

File Name: Supplementary Movie 1
Description: corresponding to Supplementary Fig. 1c. U2OS cells stably expressing nAC-GFP were stimulated by 750 nM A23187 to visualize nuclear F-actin polymerization.

File Name: Supplementary Movie 2
Description: corresponding to Supplementary Fig. 1c. Transient formation of nuclear F-actin after cell division in U2OS cells stably expressing nAC-GFP.

File Name: Supplementary Movie 3
Description: corresponding to Supplementary 1e. Distinct and transient nuclear actin filaments are detected in replicating U2OS cells stably expressing nAC-GFP.
