# Supplementary material for: Nuclear actin polymerization rapidly mediates replication fork remodeling upon stress by limiting PrimPol activity
Source: Nat Commun. 2023 Nov 28;14:7819. doi: 10.1038/s41467-023-43183-5 (PMC10684888; doi:10.1038/s41467-023-43183-5)
Supplement: Supplementary file 9 — Reporting Summary [file 41467_2023_43183_MOESM9_ESM.pdf]

nature portfolio

Corresponding author(s): Irene Chiolo, Massimo Lopes

Last updated by author(s): 20.10.2023

Reporting Summary

Nature Portfolio wishes to improve the reproducibility of the work that we publish. This form provides structure for consistency and transparency in reporting. For further information on Nature Portfolio policies, see our Editorial Policies and the Editorial Policy Checklist.

Statistics

For all statistical analyses, confirm that the following items are present in the figure legend, table legend, main text, or Methods section.

n/a	Confirmed	
☐	☒	The exact sample size (*n*) for each experimental group/condition, given as a discrete number and unit of measurement
☐	☒	A statement on whether measurements were taken from distinct samples or whether the same sample was measured repeatedly
☐	☒	The statistical test(s) used AND whether they are one- or two-sided<br>*Only common tests should be described solely by name; describe more complex techniques in the Methods section.*
☒	☐	A description of all covariates tested
☐	☒	A description of any assumptions or corrections, such as tests of normality and adjustment for multiple comparisons
☐	☒	A full description of the statistical parameters including central tendency (e.g. means) or other basic estimates (e.g. regression coefficient) AND variation (e.g. standard deviation) or associated estimates of uncertainty (e.g. confidence intervals)
☐	☒	For null hypothesis testing, the test statistic (e.g. *F*, *t*, *r*) with confidence intervals, effect sizes, degrees of freedom and *P* value noted<br>*Give P values as exact values whenever suitable.*
☒	☐	For Bayesian analysis, information on the choice of priors and Markov chain Monte Carlo settings
☒	☐	For hierarchical and complex designs, identification of the appropriate level for tests and full reporting of outcomes
☒	☐	Estimates of effect sizes (e.g. Cohen's *d*, Pearson's *r*), indicating how they were calculated

Our web collection on statistics for biologists contains articles on many of the points above.

Software and code

Policy information about availability of computer code

Data collection	A description of the individual microscope and software for the collection of DNA fiber, Metaphase, Immunofluorescence of fixed cells, live cell imaging and Electron microscopy data can be found in the Material and Methods Section of the corresponding technique.
Data analysis	Original images from Metaphase Spreads, DNA fibers, protein blot quantification, Electron microcopy were analyzed in Image J and subsequently plotted using Excel or GraphPad Prism. Image processing for live and fixed cell data of nuclear F-actin was performed with IMARIS (Bitplane), FIJI (NIH) and Photoshop CC(Adobe) Live cell imaging processing was based on Excel, MatLab (MathWorks) for MSD and for LifeAct and F-Tractin probe Zen blue software (Zeiss) was used. For length and colocalization measurements Imaris was used.

For manuscripts utilizing custom algorithms or software that are central to the research but not yet described in published literature, software must be made available to editors and reviewers. We strongly encourage code deposition in a community repository (e.g. GitHub). See the Nature Portfolio guidelines for submitting code & software for further information.

Data

Policy information about availability of data

All manuscripts must include a data availability statement. This statement should provide the following information, where applicable:

- Accession codes, unique identifiers, or web links for publicly available datasets
- A description of any restrictions on data availability
- For clinical datasets or third party data, please ensure that the statement adheres to our policy

Raw data used to build all graphs and derive statistics - as well as original, uncropped blots - are available in the provided Source data file. Microscopy images are in the range of several Terabytes and would anyway require a trained eye for interpretation. They will hence be made available upon reasonable request.

Research involving human participants, their data, or biological material

Policy information about studies with human participants or human data. See also policy information about sex, gender (identity/presentation), and sexual orientation and race, ethnicity and racism.

Reporting on sex and gender	n/a
Reporting on race, ethnicity, or other socially relevant groupings	n/a
Population characteristics	n/a
Recruitment	n/a
Ethics oversight	n/a

Note that full information on the approval of the study protocol must also be provided in the manuscript.

Field-specific reporting

Please select the one below that is the best fit for your research. If you are not sure, read the appropriate sections before making your selection.

☒ Life sciences ☐ Behavioural & social sciences ☐ Ecological, evolutionary & environmental sciences

For a reference copy of the document with all sections, see nature.com/documents/nr-reporting-summary-flat.pdf

Life sciences study design

All studies must disclose on these points even when the disclosure is negative.

Sample size	Sample size for all experiments shown (electron microscopy, n>70; DNA fibers, n>100; metaphase spreads, n>30; live cell imaging, n>8; Immunofluorescence of fixed cells, n>20) was chosen to obtain statistical power, in conformity to accepted standard sample size in a number of previous publications using the same approaches.
Data exclusions	No data were excluded from the analysis.
Replication	For all experiments, the number of biological replicates is indicated and, without any exception, reproduced trends of the representative data shown in the figures.
Randomization	Randomization does not to apply to our work which is exclusively conducted in cell lines.
Blinding	For none of the experiments in our ms, data acquisition and collection was conducted blindly, as no analysis or interpretation is doable in this phase, possibly introducing bias. Data analysis of all repetitions for Metaphase Spreading, DNA fiber analysis, Electron Microscopy and Immunofluorescence was blinded to the investigators. For life-cell imaging apporaches, all images were intrinsically unbiasedly post-processed through the same pipeline.

Behavioural & social sciences study design

All studies must disclose on these points even when the disclosure is negative.

Study description	n/a
Research sample	n/a

Sampling strategy	n/a
Data collection	n/a
Timing	n/a
Data exclusions	n/a
Non-participation	n/a
Randomization	n/a

Ecological, evolutionary & environmental sciences study design

All studies must disclose on these points even when the disclosure is negative.

Study description	n/a
Research sample	n/a
Sampling strategy	n/a
Data collection	n/a
Timing and spatial scale	n/a
Data exclusions	n/a
Reproducibility	n/a
Randomization	n/a
Blinding	n/a

Did the study involve field work? ☐ Yes ☒ No

Field work, collection and transport

Field conditions	n/a
Location	n/a
Access & import/export	n/a
Disturbance	n/a

Reporting for specific materials, systems and methods

We require information from authors about some types of materials, experimental systems and methods used in many studies. Here, indicate whether each material, system or method listed is relevant to your study. If you are not sure if a list item applies to your research, read the appropriate section before selecting a response.

Materials & experimental systems

n/a	Involved in the study
☐	☒ Antibodies
☐	☒ Eukaryotic cell lines
☒	☐ Palaeontology and archaeology
☒	☐ Animals and other organisms
☒	☐ Clinical data
☒	☐ Dual use research of concern
☒	☐ Plants

Methods

n/a	Involved in the study
☒	☐ ChIP-seq
☒	☐ Flow cytometry
☒	☐ MRI-based neuroimaging

Antibodies

Antibodies used

Mouse anti-PCNA (PC10) Santa CruzBiotechnology, Cat # sc-56;
Rabbit anti-Histone H3, Abcam, Cat # ab1791;
Mouse anti-beta-Actin (IgG1 isotype), Sigma-Aldrich, Cat # A5441;
Recombinant rabbit anti-SMARCAL1 [EPR23912-44], Abcam, Cat # ab259972;
Rabbit anti-RAD51, Bio Academia, Cat # 70-001;
Rat anti-Primpol, kind gift of Juan Méndez lab (González-Acosta 2021);
anti-RPA2 [9H8], Abcam, Cat # ab2175;
anti-Tubulin [B-5-1-2], Sigma-Aldrich, Cat # T6074;
Recombinant rabbit monoclonal anti-ATR pT1989 [EPR21991], Abcam, Cat # AB227851;
rabbit anti-ATR, Thermo Fisher Scientific, Cat # A300-137A;
Rabbit monoclonal anti-CHK1 pS345 [133D3], Cell Signaling Technology, Cat # 2348;
Mouse monoclonal anti-CHK1 [G-4], Santa Cruz Biotechnology, Cat # sc-8408;
Rabbit polyclonal anti-TFIIH [S-19], Santa Cruz Biotechnology, Cat # sc-293;
Rabbit polyclonal anti-RECQ1, Novus Biologicals, Cat # NB100-618;
Mouse monoclonal anti-GAPDH [6C5], Millipore, Cat # MAB374
Rabbit monoclonal anti-Arp3 [EPR10428(B)] , AbcamCat, # ab151729;
Rat monoclonal anti-BrdU (CldU) [BU1/75 (ICR1)], Abcam, Cat # ab6326;
Mouse anti-BrdU (IdU) [Clone B44 (RUO (GMP))], Becton Dickinson, Cat # 347580;
Goat anti-mouse-AlexaFluor488 (IgG,) H+L secondary antibody, Thermo Fisher Scientific, Cat # A-11001;
Donkey anti-rat-Cy3 polyclonal secondary antibody, Jackson ImmunoResearch via LubioScience, Cat # 712-166.153;
Rabbit polyclonal anti-FLAG, Sigma-Aldrich, Cat # F7425;
Mouse monoclonal anti-gH2AX Ser139 [clone JBW301], Millipore, Cat # 05-636;
RFP–PCNA chromobody, Chromotek, Cat # ccr;
NLS–GFP–actin chromobody, Chromotek, Cat # acgn;
anti-SMC1 polyclonal, ThermoFisher, Cat # PA5-29122;
anti-rabbit-HRP linked, VWR, Cat # NA934;
anti-mouse-HRP linked, VWR, Cat # NA931.

Validation

The PCNA antibody was previously validated using siRNA and shRNA as stated by the manufacturer: https://datasheets.scbt.com/sc-56.pdf.

The H3 has been validated using a peptide block as shown on the manufacturer's website: https://www.abcam.com/products/primary-antibodies/histone-h3-antibody-nuclear-marker-and-chip-grade-ab1791.html.

The beta-Actin antibody has been tested in this study Lessard, J., Cell Motil. Cytoskel., 10, 349 (1988) as indicated on the manufacturer's website: https://www.sigmaaldrich.com/deepweb/assets/sigmaaldrich/product/documents/296/386/a5441dat.pdf.

The Smarcal1 antibody was validated using an isotype control on flow cytometry as stated on the manufacturer's website: https://www.abcam.com/products/primary-antibodies/smarcal1-antibody-epr23912-44-ab259972.html#lb. The antibody was further validated in house using an siRNA directed against Smarcal1.

The Rad51 antibody was validated in house using siRNA in immunofluorescence and western blot approaches. The antibody was further tested in many studies, some of them indicated on the manufacturer's website: https://www.bioacademia.co.jp/en/html/upload/save_image/E70-001%20anti-Rad51(human)antibody(rabbit-serum).pdf.

Rat anti-Primpol antibody was validated in house for Western blotting using siRNA .

The RPA antibody was validated in IHC-P, Flow Cyt, WB and tested in Human samples and cited in 182 publications as stated by the manufacturer: https://www.abcam.com/en-lu/products/primary-antibodies/anti-rpa32-rpa2-antibody-9h8-ab2175.

The Tubulin antibody was validated via knock out western blotting studies as claimed on the manufacturer's website: https://www.sigmaaldrich.com/CH/de/product/sigma/t6074?gclid=CjwKCAjwysipBhBXEiwApJOcu7IIHlFYe9j42OxfPxeGsSU5sMEHC97oIgosIngjPuj37NH1EBL_WhoC7jkQAvD_BwE.

The ATR pT1989 antibody was validated for Western blotting using a phosphatase and a ATR non-phospho peptide: https://www.abcam.com/products/primary-antibodies/atr-phospho-t1989-antibody-epr21991-ab223258.html.

The ATR antibody was validated in Immunoprecipitation Western blotting using an IgG control as claimed on the manufacturer's

website: https://www.thermofisher.com/antibody/product/ATR-Antibody-Polyclonal/A300-137A.

The CHK1 pS345 antibody was validated for Western blotting and Immunofluorescence using untreated versus UV-irradiated cells as shown on the manufacturer's website: https://www.cellsignal.com/products/primary-antibodies/phospho-chk1-ser345-133d3-rabbit-mab/2348. The antibody was moreover cited in over 100 publications in the past and was validated using an ATR inhibitor and a ETAA1/TOBP1 - deficient cell line by many labs and in house.

The CHK1 antibody was cited in 813 publications and a validation in intracellular FCM analysis using an IgG control is displayed on the manufacturer's website: https://www.scbt.com/p/chk1-antibody-g-4.

The TFIIH antibody has been tremendously published: https://www.scbt.com/p/tfiih-p89-antibody-s-19.

The RECQ1 antibody was validated in house using siRNA or shRNA in Western blotting as shown in our references on the manufacturer's website: https://www.novusbio.com/products/recq1-antibody_nb100-618#PublicationSection.

The GAPDH antibody is a well published and extensively characterized monoclonal antibody and has been validated for use in ELISA, IP, IC, IF, IH & WB as claimed by the manufacturer: https://www.merckmillipore.com/CH/de/product/Anti-Glyceraldehyde-3-Phosphate-Dehydrogenase-Antibody-clone-6C5,MM_NF-MAB374?ReferrerURL=https%3A%2F%2Fwww.google.com%2F#.

The Arp-3 antibody has been published in these two references as stated by Abcam: https://www.abcam.com/products/primary-antibodies/arp3-antibody-epr10428b-ab151729.html.

The rat BrdU antibody has been extensively published and validated amongst other means with a BrdU-negative control sample in Immunofluorescence (see manufacturer's website: https://www.abcam.com/products/primary-antibodies/brdu-antibody-bu175-icr1-proliferation-marker-ab6326.html).

The mouse BrdU antibody has been extensively used in the field and validated using BrdU-negative control conditions: https://www.bdbiosciences.com/content/bdb/paths/generate-tds-document.ch.347580.pdf.

The goat-anti-mouse A488 secondary is extensively used in the field and has been tested for immunofluorescence in controls lacking the primary antibody: https://www.thermofisher.com/antibody/product/Goat-anti-Mouse-IgG-H-L-Cross-Adsorbed-Secondary-Antibody-Polyclonal/A-11001.

The donkey anti-rat Cy3 antibody is heavily described in the literature and is commonly tested with no primary antibody controls: https://www.jacksonimmuno.com/catalog/products/712-166-153

The specificity of the anti-Flag antibody was validated in our ms using the Flag-NLS-ActinR62D mutant construct. More citations and infos can be found here: https://www.sigmaaldrich.com/CH/de/product/sigma/f7425. Also see this citation for further validation: https://www.sciencedirect.com/science/article/pii/S109727652030647X?via%3Dihub.

The anti-phospho-Histone H2A.X (Ser139) antibody has been extensively used by our lab and others and was validated using treated versus non-treated controls and applied in Western blotting to a S139-unphosphorylatable RPE-1 cell line carrying a point-mutation. More info here: https://www.merckmillipore.com/CH/de/product/Anti-phospho-Histone-H2A.X-Ser139-Antibody-clone-JBW301,MM_NF-05-636-I?ReferrerURL=https%3A%2F%2Fwww.google.com%2F#.

The PCNA chromobody was tested for its expected (based on previous data from fixed cells stained e.g. with a PCNA antibody) expression and distribution throughout the live S-phase of the cell cycle as shown by the manufacturer: https://www.ptglab.com/products/Cell-Cycle-Chromobody-plasmid-TagRFP-ccr.htm

The NLS-GFP-actin chromobody was validated for expected expression/distribution in our ms using calcium ionophore A23187 or during mitotic exit. Please see the manufacturer's website for further validation: https://www.ptglab.com/products/Nuclear-Actin-Chromobody-plasmid-TagGFP-acg-n.htm#publications.

The SMC1 antibody was veryfied by knockdown as stated on the manufacturer's website: https://www.thermofisher.com/antibody/product/SMC1-Antibody-Polyclonal/PA5-29122.

Eukaryotic cell lines

Policy information about cell lines and Sex and Gender in Research

Cell line source(s)	Human U2OS (ATCC) and RPE-1, HEK293T cell lines from the IMCR were used for this study. Nuclear-actin-chromobody(nAC-GFP)stable U2OS cells and stable doxycycline-inducible NLS-BFP-Actin or NLS-BFP-ActinR62D cells were kindly provided by Dr. Robert Grosse, University of Freiburg (Germany).
Authentication	None of the cell lines were authenticated in house for this manuscript.
Mycoplasma contamination	U2OS and RPE-1 cells are subject to our regular mycoplasma testing in house and HEK293T cells tested negative for this study. All other cell lines were not tested for mycoplasma for this study.
Commonly misidentified lines (See ICLAC register)	No commonly misidentified cell lines were used in this study.

Palaeontology and Archaeology

Specimen provenance	n/a
Specimen deposition	n/a
Dating methods	n/a

☐ Tick this box to confirm that the raw and calibrated dates are available in the paper or in Supplementary Information.

Ethics oversight	n/a

Note that full information on the approval of the study protocol must also be provided in the manuscript.

Animals and other research organisms

Policy information about studies involving animals; ARRIVE guidelines recommended for reporting animal research, and Sex and Gender in Research

Laboratory animals	n/a
Wild animals	n/a
Reporting on sex	n/a
Field-collected samples	n/a
Ethics oversight	n/a

Note that full information on the approval of the study protocol must also be provided in the manuscript.

Clinical data

Policy information about clinical studies

All manuscripts should comply with the ICMJE guidelines for publication of clinical research and a completed CONSORT checklist must be included with all submissions.

Clinical trial registration	n/a
Study protocol	n/a
Data collection	n/a
Outcomes	n/a

Dual use research of concern

Policy information about dual use research of concern

Hazards

Could the accidental, deliberate or reckless misuse of agents or technologies generated in the work, or the application of information presented in the manuscript, pose a threat to:

No	Yes	
☒	☐	Public health
☒	☐	National security
☒	☐	Crops and/or livestock
☒	☐	Ecosystems
☒	☐	Any other significant area

Experiments of concern

Does the work involve any of these experiments of concern:

No	Yes	
☒	☐	Demonstrate how to render a vaccine ineffective
☒	☐	Confer resistance to therapeutically useful antibiotics or antiviral agents
☒	☐	Enhance the virulence of a pathogen or render a nonpathogen virulent
☒	☐	Increase transmissibility of a pathogen
☒	☐	Alter the host range of a pathogen
☒	☐	Enable evasion of diagnostic/detection modalities
☒	☐	Enable the weaponization of a biological agent or toxin
☒	☐	Any other potentially harmful combination of experiments and agents

Plants

Seed stocks	n/a
Novel plant genotypes	n/a
Authentication	n/a

ChIP-seq

Data deposition

☐ Confirm that both raw and final processed data have been deposited in a public database such as GEO.

☐ Confirm that you have deposited or provided access to graph files (e.g. BED files) for the called peaks.

Data access links<br>*May remain private before publication.*	*For "Initial submission" or "Revised version" documents, provide reviewer access links. For your "Final submission" document, provide a link to the deposited data.*
Files in database submission	*Provide a list of all files available in the database submission.*
Genome browser session<br>(e.g. UCSC)	*Provide a link to an anonymized genome browser session for "Initial submission" and "Revised version" documents only, to enable peer review. Write "no longer applicable" for "Final submission" documents.*

Methodology

Replicates	*Describe the experimental replicates, specifying number, type and replicate agreement.*
Sequencing depth	*Describe the sequencing depth for each experiment, providing the total number of reads, uniquely mapped reads, length of reads and whether they were paired- or single-end.*
Antibodies	*Describe the antibodies used for the ChIP-seq experiments; as applicable, provide supplier name, catalog number, clone name, and lot number.*
Peak calling parameters	*Specify the command line program and parameters used for read mapping and peak calling, including the ChIP, control and index files used.*
Data quality	*Describe the methods used to ensure data quality in full detail, including how many peaks are at FDR 5% and above 5-fold enrichment.*
Software	*Describe the software used to collect and analyze the ChIP-seq data. For custom code that has been deposited into a community repository, provide accession details.*

Flow Cytometry

Plots

Confirm that:

- ☐ The axis labels state the marker and fluorochrome used (e.g. CD4-FITC).
- ☐ The axis scales are clearly visible. Include numbers along axes only for bottom left plot of group (a 'group' is an analysis of identical markers).
- ☐ All plots are contour plots with outliers or pseudocolor plots.
- ☐ A numerical value for number of cells or percentage (with statistics) is provided.

Methodology

Sample preparation	*Describe the sample preparation, detailing the biological source of the cells and any tissue processing steps used.*
Instrument	*Identify the instrument used for data collection, specifying make and model number.*
Software	*Describe the software used to collect and analyze the flow cytometry data. For custom code that has been deposited into a community repository, provide accession details.*
Cell population abundance	*Describe the abundance of the relevant cell populations within post-sort fractions, providing details on the purity of the samples and how it was determined.*
Gating strategy	*Describe the gating strategy used for all relevant experiments, specifying the preliminary FSC/SSC gates of the starting cell population, indicating where boundaries between "positive" and "negative" staining cell populations are defined.*

☐ Tick this box to confirm that a figure exemplifying the gating strategy is provided in the Supplementary Information.

Magnetic resonance imaging

Experimental design

Design type	*Indicate task or resting state; event-related or block design.*
Design specifications	*Specify the number of blocks, trials or experimental units per session and/or subject, and specify the length of each trial or block (if trials are blocked) and interval between trials.*
Behavioral performance measures	*State number and/or type of variables recorded (e.g. correct button press, response time) and what statistics were used to establish that the subjects were performing the task as expected (e.g. mean, range, and/or standard deviation across subjects).*

Acquisition

Imaging type(s)	*Specify: functional, structural, diffusion, perfusion.*
Field strength	*Specify in Tesla*
Sequence & imaging parameters	*Specify the pulse sequence type (gradient echo, spin echo, etc.), imaging type (EPI, spiral, etc.), field of view, matrix size, slice thickness, orientation and TE/TR/flip angle.*
Area of acquisition	*State whether a whole brain scan was used OR define the area of acquisition, describing how the region was determined.*

Diffusion MRI ☐ Used ☐ Not used

Preprocessing

Preprocessing software	*Provide detail on software version and revision number and on specific parameters (model/functions, brain extraction, segmentation, smoothing kernel size, etc.).*
Normalization	*If data were normalized/standardized, describe the approach(es): specify linear or non-linear and define image types used for transformation OR indicate that data were not normalized and explain rationale for lack of normalization.*
Normalization template	*Describe the template used for normalization/transformation, specifying subject space or group standardized space (e.g. original Talairach, MNI305, ICBM152) OR indicate that the data were not normalized.*
Noise and artifact removal	*Describe your procedure(s) for artifact and structured noise removal, specifying motion parameters, tissue signals and physiological signals (heart rate, respiration).*

Volume censoring | Define your software and/or method and criteria for volume censoring, and state the extent of such censoring.

Statistical modeling & inference

Model type and settings | *Specify type (mass univariate, multivariate, RSA, predictive, etc.) and describe essential details of the model at the first and second levels (e.g. fixed, random or mixed effects; drift or auto-correlation).*

Effect(s) tested | *Define precise effect in terms of the task or stimulus conditions instead of psychological concepts and indicate whether ANOVA or factorial designs were used.*

Specify type of analysis: ☐ Whole brain ☐ ROI-based ☐ Both

Statistic type for inference | *Specify voxel-wise or cluster-wise and report all relevant parameters for cluster-wise methods.*

(See Eklund et al. 2016)

Correction | *Describe the type of correction and how it is obtained for multiple comparisons (e.g. FWE, FDR, permutation or Monte Carlo).*

Models & analysis

n/a	Involved in the study
☐	☐ Functional and/or effective connectivity
☐	☐ Graph analysis
☐	☐ Multivariate modeling or predictive analysis

Functional and/or effective connectivity | *Report the measures of dependence used and the model details (e.g. Pearson correlation, partial correlation, mutual information).*

Graph analysis | *Report the dependent variable and connectivity measure, specifying weighted graph or binarized graph, subject- or group-level, and the global and/or node summaries used (e.g. clustering coefficient, efficiency, etc.).*

Multivariate modeling and predictive analysis | *Specify independent variables, features extraction and dimension reduction, model, training and evaluation metrics.*
